# An Authentication Survey on Retail Seafood Products Sold on the Bulgarian Market Underlines the Need for Upgrading the Traceability System

**DOI:** 10.3390/foods12051070

**Published:** 2023-03-02

**Authors:** Lara Tinacci, Deyan Stratev, Mariyana Strateva, Georgi Zhelyazkov, Ralica Kyuchukova, Andrea Armani

**Affiliations:** 1 Department of Veterinary Sciences, University of Pisa, Via delle Piagge 2, 56124 Pisa, Italy; 2 Department of Food Quality and Safety and Veterinary Legislation, Faculty of Veterinary Medicine, Trakia University, 6000 Stara Zagora, Bulgaria; 3 Department of Veterinary Anatomy, Histology and Embryology, Faculty of Veterinary Medicine, Trakia University, 6000 Stara Zagora, Bulgaria; 4 Department of Animal Husbandry—Non-Ruminants and Other Animals, Faculty of Agriculture, Trakia University, 6000, Stara Zagora, Bulgaria

**Keywords:** seafood labeling, authenticity, mislabeling, DNA barcoding, PCR–RFLP analysis seafood, marking

## Abstract

Economically motivated or accidental species substitutions lead to economic and potential health damage to consumers with a loss of confidence in the fishery supply chain. In the present study, a three–year survey on 199 retail seafood products sold on the Bulgarian market was addressed to assess: (1) product authenticity by molecular identification; (2) trade name compliance to the list of official trade names accepted in the territory; (3) adherence of the list in force to the market supply. DNA barcoding on mitochondrial and nuclear genes was applied for the identification of whitefish (WF), crustaceans (C) and mollusks (cephalopods—MC; gastropods—MG; bivalves—MB) except for *Mytilus* sp. products for which the analysis was conducted with a previously validated RFLP PCR protocol. Identification at the species level was obtained for 94.5% of the products. Failures in species allocation were reconducted due to low resolution and reliability or the absence of reference sequences. The study highlighted an overall mislabeling rate of 11%. WF showed the highest mislabeling rate (14%), followed by MB (12.5%), MC (10%) and C (7.9%). This evidence emphasized the use of DNA–based methods as tools for seafood authentication. The presence of non–compliant trade names and the ineffectiveness of the list to describe the market species varieties attested to the need to improve seafood labeling and traceability at the national level.

## 1. Introduction

Global fish consumption has grown steadily in the last five decades, driven by a combination of population growth, rising incomes and changes in food habits, as well as strong expansion in fish production [1]. Aquatic foods are increasingly recognized for their key role in food security and global nutrition. In addition, they represent an invaluable source of income and employment with a direct impact on the livelihoods of a substantial percentage of the population, mainly in Asia and Africa. According to FAO, the amount of seafood destined for human consumption in 2020 was estimated to be 20.2 kg per capita, doubling the average of 9.9 kg per capita noted in 1960. While a plateau has been reached for the volumes of aquatic products obtained from fishing activities, this trend is set to grow further, mainly due to the expansion and modernization of aquaculture systems. The increase in per capita consumption is also favorably influenced by the speeding–up of the supply chain and the improvement of transport logistics, which have led to the exponential expansion of the global product offer [1]. The European Union (EU) holds a considerable position in this scenario, with consumption estimates of around 25 kg per capita per year [2]. However, a significant variation between Member States exists. Seafood prices, purchasing ability of the consumer and net income, and culturally–driven dietary preference are the factors most frequently affecting the variability of consumption at EU and international levels [3].

In Bulgaria, apparent domestic seafood consumption appears to be limited, ranging from 5.3 kg to 7.5 kg per capita per year, and an average value of 6 kg per capita, according to EUMOFA and the Bulgarian Ministry of Agriculture for the years 2019–2020 [2,4]. In addition, according to data published by the EU Commission on consumer habits regarding fishery and aquaculture products for the year 2021, the consumption rate generally does not exceed a monthly frequency [5]. Evidence of this limited consumption is also attested by a consumer survey conducted in 2018 by Stancheva [6], from which emerged that seafood products have an average consumption rate not exceeding 1–2 times per month by about the 46% of the respondents, while only 26% declared weekly consumption. The low buying appetite towards seafood is generally due to (1) the consumer’s lack of perception of the benefits of a regular consumption and (2) the medium/high price of seafood [7]. These aspects could therefore constitute major limiting factors to the expansion of seafood market demand in Bulgaria.

However, despite the unchanged consumption of fresh water and marine seafood from both inland waters and the coast of the Black Sea, an increase in fresh, chilled, frozen and variously processed marine seafood imports (fish, crustaceans and mollusks) of EU and extra–EU origin on the Bulgarian market has been recently reported [8]. This evidence was also previously highlighted in a survey conducted in 2019, in which the presence of imported seafood, especially at the large retail level, was described as mostly pre–packaged fresh and frozen [9].

It is well known that globalization and the complexity of the supply chain, together with the distribution system, can potentially expose the seafood market to an increased opportunity for the perpetration of deceptive behaviors [10,11]. Fraudulent incidents are frequently described, leading to misdescriptions, mislabeling and economically motivated species substitutions, resulting in consumer economic damage and loss of confidence in the sector chain. These incidents could potentially include health risks whenever substitution events include the illicit presence of toxic species or the omission of allergens [11,12,13].

To ensure safety, transparency, and fair trading, which are founding principles of EU food law [14,15], specific provisions have been issued for the labeling and presentation of the sale of fishery products by EU Regulation No.1379/2013 [16]. Pursuant to this regulation, the Member States are delegated to publish and update a list reporting the official seafood trade names corresponding to the species’ scientific names accepted in the national territories for product sales. According to the current EU legislation, the responsibility for the correctness of the information of the product to the consumer lies directly with the food business operator who prepares the product for sale; the organization of official controls on labeling is entrusted to a central competent authority identified by each member state [15,16,17]. Specifically, in Bulgaria, the responsibility for official controls in this area is delegated to the Bulgaria Food Safety Agency (BFSA; http://www.bfsa.bg, accessed on 30 October 2022).

Nonetheless, fraudulent incidents in the seafood supply chain are still well documented both at the international and EU level [11,12,13], although data as regards labeling compliance and mislabeling seafood rates in Central–Eastern and Eastern European countries are rarely reported [16]. In this respect, the regulation also promotes control measures aimed at verifying the identity of products through the use of available technology, including DNA testing, to deter fraudulent substitution practices. Several DNA–testing techniques have been successfully applied in market surveys to verify the labeling compliance of commercial seafood collected at retail. Particularly, sequencing–based methods like FINS and DNA barcoding based on the analysis of mitochondrial (*COI*, *cytb*; *16S rRNA*) targets, are recognized as valuable tools for seafood species identification. Nuclear targets (*rhodopsin*, Phosphoenolpyruvate Carboxykinase (*PEPCK*), and Polyphenolic Adhesive Protein (*PAP*)) have been additionally selected in the presence of introgression or hybridization phenomena [18,19,20]. Additionally and alternatively, PCR–restriction fragment length polymorphism (RFLP), amplified fragment length polymorphism (AFLP), or single–stranded conformational polymorphism (SSCP) on both mitochondrial and nuclear targets have been applied as targeted methods to design genus or species–specific assays [19,20]. In this regard, the validity of an RFLP protocol on the PAP gene for species–specific discrimination of bivalve mollusks—mussels—belonging to the genus *Mytilus* sp., has been confirmed [21].

The exposure of the Bulgarian market to fraudulent phenomena was first documented in a pilot study conducted in 2017 [22]. In that survey, a DNA–barcoding approach applied to assess the product’s authenticity highlighted an overall substitution rate of 17.7%. The species substitutions were equally distributed between unprocessed and processed products (smoked and marinated products). Particularly, five plausibly deceptive incidents for economic gain were finally pointed out, two of which consisted in the replacement of *Gadus chalcogramma* (Alaska pollock) with *Pangasianodon hypophthalmus* (striped catfish) and the remaining three cases in the replacement of *Illex argentinus* (Argentine squid) with *Dosidicus gigas* (Humboldt squid). The remaining mislabeling incidents, consisting of species substitution between species of similar commercial value, were linked the improper training of the operators in species morphological identification [22]. Furthermore, the study highlighted the urgency of the revision of the Bulgarian official list of seafood trade names. This evidence was reaffirmed in a further study on the validity and accuracy of the new official Bulgarian list of seafood trade and the list evolution and adherence to the Bulgarian market’s trend [23].

Considering the evidence acquired in the pilot study on products belonging to two taxonomic classes (fish and gastropod mollusks) purchased at retail in a single city (Stara Zagora) [22], a three–year survey was conducted for the assessment of the authenticity of seafood products belonging to five distinct taxonomic classes, purchased in retail settings in four different cities across the country. The products’ molecular identification to verify the compliance of the labeled species designations was conducted by means of DNA barcoding and PCR–RFLP based on mitochondrial and nuclear markers. Furthermore, the labeled designations were checked against the Bulgarian official list of seafood trade names to verify the use of authorized commercial designations. Finally, the comparison of the labeled designation with the accepted designation in force was performed to verify the adherence of the official list to the products basket available at retail.

## 2. Materials and Methods

### 2.1. Samples Collection: Sampling Strategy and Products Variety

In the present study, the sampling plan for selecting the seafood categories was structured by considering the characteristics of the Bulgarian market in terms of production, imports and exports [8] and the most frequent seafood fraud incidents reported in a previous study [22] and at the EU level.

Within the fish taxonomical class, the sampling was targeted on white fish (WF) since Tinacci et al. [22] observed that deliberate substitutions mainly involved Alaskan pollock (*Gadus chalcogrammus*) products. This choice was also performed in response to clear evidence of deliberate substitution phenomena involving valuable WF, particularly those belonging to the Gadidae and Merluccidae families, both at the international and EU levels [12,24,25,26,27]. In addition, in a nationwide survey conducted on the Bulgarian fish retail market, Gadidae and Merluccidae were reported to be among the products (frozen and filleted) most frequently found on sale, especially in large retail settings [9]. The survey also revealed the association of a few generic commercial designations for a considerable number of species belonging to the two families characterized by heterogeneous commercial values. This aspect was considered by the authors as favoring consumers’ confusion on fish value and market exposure to deceitful incidents for economic gain. [9].

Within cephalopods class, the number of products was mainly selected in relation to the results of the pilot study and the sampling was primarily targeted at squid products, for which three substitutions were shown in the limited sampling of products under analysis (N = 11) [22]. Moreover, the sampling was also extended to crustaceans, mollusks bivalves, and gastropods classes by virtue of the increased interest in the Bulgarian market, showing crustaceans to be second in terms of nationwide production and import, followed by mollusk categories [8]. For gastropod mollusks (MG), the sampling was deliberately reduced as, according to bibliographic data, their consumption is mostly limited to the *Rapana venosa* species [28]. The products were purchased from large distribution retailers in four different towns (Stara Zagora, Shumen, Varna and Dobrich) from March 2019 to July 2021, and the sampling resulted in the collection of 199 seafood products, including whitefish (WF; N = 100), cephalopod mollusks (MC; N = 40), crustaceans (C; N = 38), mollusks bivalves (MB; N = 16), and mollusks gastropods (MG; N = 5). Therefore, the different proportion of products collected for the five product categories (WF = 100, 50.3%; MC = 40, 20.1%; C = 38, 19.1%; MB = 16, 8.0%; MG; N = 5, 2.5%) more or less reflects the weight of the various categories on the national market.

The products were transferred to the Department of Food Quality and Safety (Faculty of Veterinary Medicine, Trakia University). Each product was classified according to Regulation (EC) No. 852/2004 [29] as unprocessed (fresh, frozen, beheaded, cleaned and filleted) or processed (marinated and precooked), then provided with a progressive numerical code, which was recorded in a single file together with labeling information (Table S1). Finally, 1–5 g of muscle tissue were collected, dehydrated by means of 95% ethanol and sent to FishLab (Department of Veterinary Science, University of Pisa) for molecular identification.

### 2.2. DNA Extraction, Molecular Target Selection, Amplification, and Sequencing

Total DNA extraction from each dehydrated tissue sample was performed according to the salting–out procedure proposed by Armani et al. [30], starting from 50 to 100 mg of tissue. Final DNA concentrations and quality were checked with a Nanodrop ND–1000 spectrophotometer (NanoDrop Technologies, Wilmington, DE, USA) according to the manufacturer guidelines and absorbance ratios A260/A280 > 2.0 and A260/A230 >1.8 were set as minimum values of nucleic acid purity. 

The selection of the molecular target followed the decisional procedure (decision tree) proposed by Tinacci et al. [31]. A fragment of 655–658 bp of the Cytochrome C oxidase subunit I (*COI*) gene was chosen as an elective target for species identification for all the product categories. Additionally, two mitochondrial gene coding for the enzyme cytochrome b and 16S ribosomal RNA (*cytb* and *16SrRNA*) and one nuclear gene coding for the enzyme Phosphoenolpyruvate Carboxykinase (*PEPCK*) were applied in the study to enhance the discriminatory molecular ability when the elective target alone failed to achieve species identification according to the seafood product’s taxonomical class under analysis. Products labeled as *Mytilus* sp. (Table S1) were only tested by the PCR–RFLP technique for the analysis of a non–repetitive region of the nuclear Polyphenolic Adhesive Foot Protein (*PAP*) gene as described in Giusti et al. [21].

Details on elective and additional targets, primer pairs and amplification protocol applied in the study are summarized in Table 1.

After amplification, 5 µL of each PCR product was checked on a 1.8% agarose gel previously stained with GelRed™ Nucleic Acid Gel Stain (Biotium, Hayward, CA, USA). The presence of the expected amplicon and the final concentration was verified by comparison with the standard molecular marker SharpMass™50–DNA (Euroclone Spa, Pero; Milano, Italy). A concentration equal to or greater than 5 μg/μL of PCR product was set as a threshold value for subsequent sequencing reaction according to the sequencing laboratory operative procedures and sent to Eurofins Genomics laboratories (Eurofins Genomics, Ebersberg, Germany).

### 2.3. Sequences Analysis and Molecular Identification

The obtained sequences were aligned and edited with Clustal W in BioEdit version 7.0.9 [38], and the final sequences were queried against the reference sequences available in GenBank (http://www.ncbi.nlm.nih.gov, accessed on 30 July 2022 ) and, in case of *COI*, the BOLD (http://www.boldsystems.org/, accessed on 30 July 2022) databases. The species was finally allocated by setting target–specific identity score cut–offs as specified below. For the *COI* and *cytb* targets, a top match identity score >98% was applied for species allocation [31,39]; for the *16SrRNA* and *PEPCK* targets, an identity score of 100% was defined as the cut–off threshold for species identification [31,40,41]. Furthermore, for species allocation with the additional targets, the Neighbor–joining method [42] and Kimura 2–parameter model [43] with 1000 bootstrap re–samplings were applied to infer distance–based dendrograms in MEGA–11 [44].

### 2.4. Assessment of Products Labeling Compliance and Mislabeling Rate

The information reported on the labels was verified against the mandatory requirements of Regulation (EU) No. 1379/2013 [16]. Specifically, for each product included in the scope of Article 35, commercial designation of the species and its scientific name, an indication of production method, catch or farming area for aquaculture products, fishing method and thawing before the sale were considered. The scientific names were verified by consulting the official databases listed in Article 37 of the Regulation (FAO Sealife base; FAO Fishbase, World Register of Marine Species (WorMS) and ASFIS databases). Thus, a direct comparison between the molecular results and the labeled scientific names was performed. The products were declared non–compliant if the molecularly identified species did not match the scientific names labeled on the product.

Furthermore, the adherence of the labeled designations against the list of accepted scientific names in force during the sampling period, included in Ordinance no. 4 of 13.01.2006 [45], was verified. The labeled designations were finally compared with the new list, published with Ordinance No. 13 of 30.11.2021 [46], published after the completion of the sampling phase. The comparison with both lists was made to assess the evolution of the list of official designations in terms of adherence to the Bulgarian market supply.

## 3. Results and Discussion

### 3.1. Distribution of Product Variety Resulting from Sampling 

The definition of the sampling strategy should be based on a preliminary study of the different product categories available on the market to better define the representative sample size for each taxonomical class [12]. Thus, the sample products collected and analyzed in this study reflect the weight of the different seafood taxonomical classes and products as assessed in the preliminary sampling strategy (see Section 2.1).

**Figure 1:**
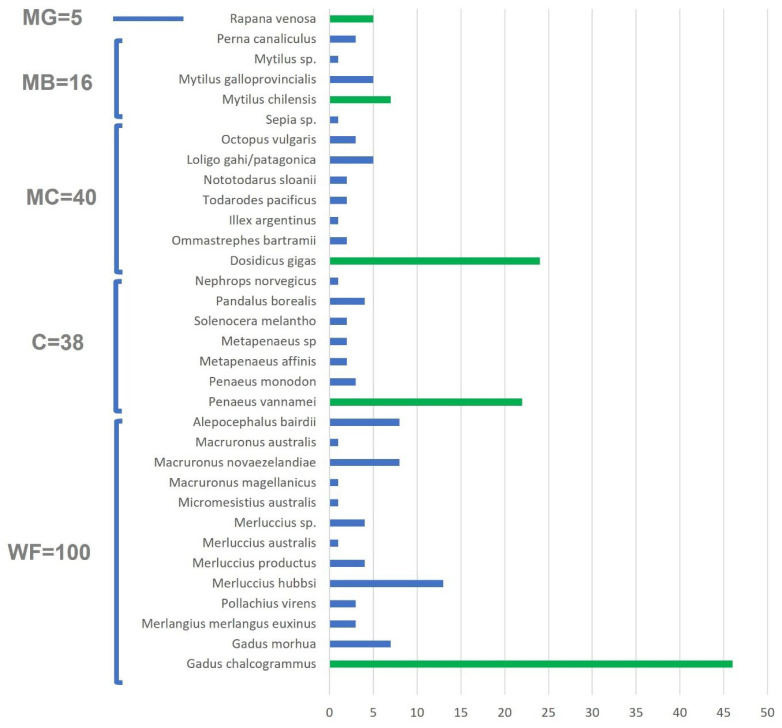
The number of seafood products collected during the sampling period at retail outlets per species declared on the label within each seafood taxonomical class (WF—fish; C—crustaceans; MC—cephalopods; MB—bivalve mollusks; MG—gastropods. In green, for each taxonomical class, the species most commonly encountered during sampling.

The study highlighted the availability at large retail levels of a fairly limited variety of species (Figure 1) in agreement with several findings from an extensive survey conducted in 2019 on the Bulgarian seafood market, both at the local and large retail levels, to assess the diversity of available retail fish products [9]. When limiting the comparison to retail data, the survey had already revealed increased attention towards marine imported products of Atlantic and Pacific origins. This finding showed a clear evolution of the consumption trend compared to a study conducted by Stancheva [6], from which the consumers’ preferences appeared to be mainly oriented towards local freshwater products or locally caught marine fish products in accordance with national culinary traditions.

Prominent species were immediately identifiable for each taxonomical class (WF, MC, MG, C). In detail, in WF, *G. chalcogrammus* accounted for 46% of products collected in the study, followed by Argentine hake (*Merluccius hubbsi*) (23%). The C and MC categories were led respectively by *Penaeus vannameii* and *Dosidicus gigas* representing 58% and 60% of the products, respectively. A greater equilibrium of species is observed within the MB in which the species *M. chilensis* is predominant but in balance with *M. galloprovincialis*, while the third species *Perna canaliculus*, was only occasionally available for sampling. With regard to MG, consumer interest, as previously introduced, is focused exclusively on *R. venosa*, the only species sampled for the taxonomical class and, according to Keskin et al. [47], one of the most exploited species along Black Sea coasts. 

**Figure 2:**
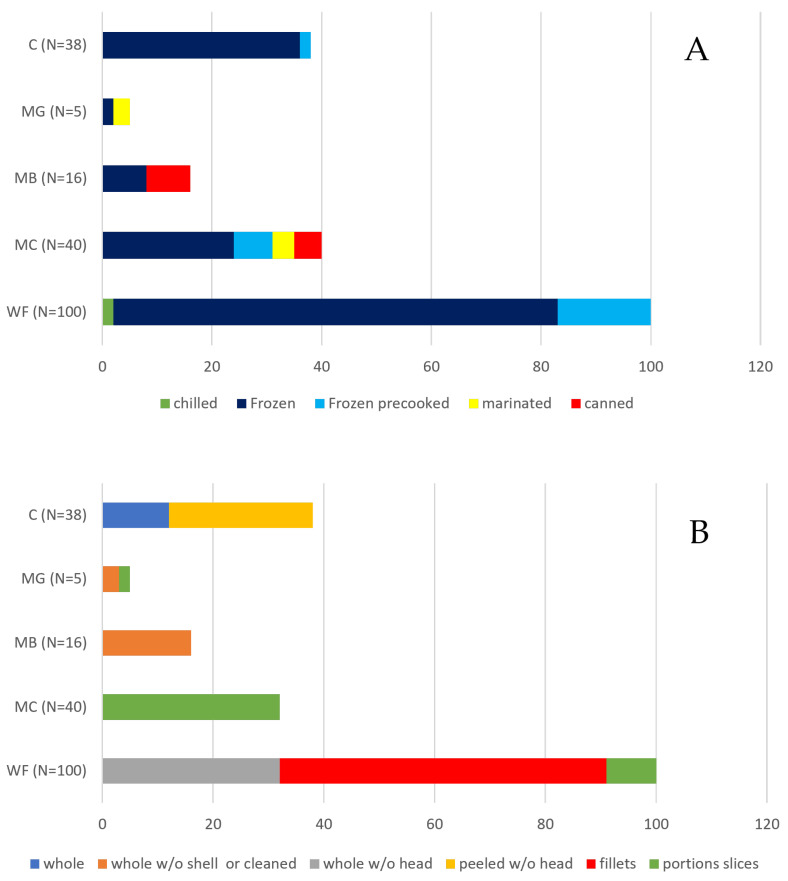
Distribution of product types within the taxonomical classes. (WF—Fish; MC—Mollusks cephalopod mollusks; MB—bivalve mollusks; MG—gastropod mollusks; C—crustaceans. (**A**) Distribution of products types classified according to Regulation (EC) No. 852/2004 [29]. (**B**) distribution of product types classified according to the type of presentation for sale (without previous preparation, post evisceration, post cutting).

Overall, 76.4% (N = 152) of the samples were made of unprocessed products (75.4% frozen and 1% chilled) and the remaining 23.6 % (N = 47) of distinct types of processed products (14% precooked frozen, 5.0% marinated and 4.5% canned) with different frequencies among the categories included in the study (Figure 2A).

A different presentation of the products at purchase for the different taxonomic classes was observed (Figure 2B). In particular, the greatest variability was observed in WF where, however, the prevalence of variously cut products (59% in fillets and 9% in slices) compared to whole products can be observed. In 100% of the MC products, a standard level of processing was observed with the absence of whole products at retail. A similar assessment applies to MB, where 100% of the products presented for sale in whole form without shells. In MG, a distribution between whole products without shells and sliced products was observed where the aquatic organism was no longer immediately recognizable as it had been reduced to a pulp. For class C finally, a clear predominance of peeled products (68.6%, 26/38) over whole specimens (31.6%, 12/38) was observed.

Thus, according to the results, the retailers imported from EU and non–EU producers seem to almost exclusively target frozen unprocessed and precooked products and less frequently canned products, except for MB. This evidence agrees with the data collected in Todorov [8], confirming the Bulgarian large retail fishery market trades are mainly oriented towards imports of prepared (frozen cleaned, filleted or sliced) and processed (canned, marinated) products of EU and non–EU origin.

This is in agreement with the description also given by Vindigni and colleagues [48] on the type of products most imported at the EU level based on data provided by the EU Commission in 2020. It is also interesting to note the degree of preparation found not only in processed products (ready–to–eat or breaded precooked) but also in unprocessed products. This observation is consistent with that reported by Stancheva [6] regarding consumer demand for easy–to–use, fresh, frozen or canned products.

### 3.2. Molecular Identification and Its Limitations

Total DNA sample was successfully extracted from all 199 products included in the study. All the 199 DNA samples were successfully amplified producing 208 PCR products (COI N = 186; cytb N = 11; 16S rRNA N = 8; PEPCK N = 3) for further sequencing and 13 PCR products (belonging to products labeled as *Mytilus* sp.) to be analyzed by RFLP (Table S2).

**Figure 3:**
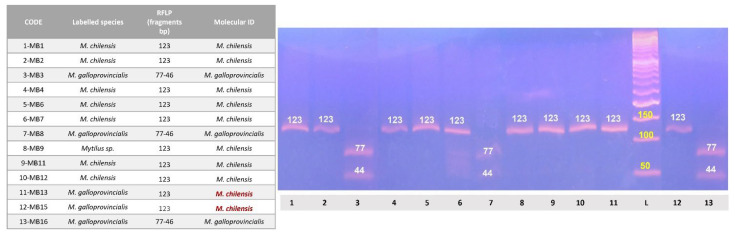
Results of PCR–RFLP analysis on DNA samples extracted from the 13 MB *Mytilus* sp. products. Electrophoretic run shows post Aci–I digestion outcomes on PCR products of *PAP* target according to Giusti et al. [21] protocol. The length (bp) of the fragments is reported above the bands. L—ladder.

All the 208 PCR products intended for post–sequencing analysis returned readable sequences. The length of the final sequences and the results of the post–sequencing analysis are shown in Table S2. Overall, a final species allocation was reached in 188 out of 199 products included in the study (94.5%) using: (1) a *COI* barcoding approach in 159 products (F = 81; MC = 39; MB = 3, C = 35); (2) or by the analysis of an additional target in 16 products (F = 11; MG = 5); (3) by the application of a PCR–RFLP protocol specifically for *Mytilus sp.* species allowed the final species allocation in 13 MB products as detailed in Figure 3. Specifically, 10 MB were finally assigned to the species *M. chilensis,* and the remaining 3 MB products (MB–3, MB–8, and MB–16) were assigned to the species *M. galloprovincialis.*

In particular, 11 WF products finally identified as *M. productus* (N = 7) and *M. gayi* (N = 4) by the analysis of *COI* and *cytb* targets (Section 3.2.1) and five MG products were finally assigned at the species level by the analysis of *COI* and *16S rRNA* targets (Section 3.2.2).

In the remaining 11 products, the analysis exclusively led to a genus–level allocation with the assignment of 8 F products to *Alepocephalus* sp. and 3 C products to *Metapenaeus* sp. (N = 2) and *Heterocarpus* sp. (N = 1). Nevertheless, all the data obtained were sufficient for the subsequent verification of labeling compliance. Major limitations to the successful species identification could be due to (1) low target barcode resolution, (2) the low reliability of reference sequences or (3) the absence of reference sequences for the comparative analysis, as already highlighted by Fernandes et al. [18].

#### 3.2.1. Low Target Barcode Resolution

Low target barcode resolution is typically described in recently diverged and/or closely related species that are geographically isolated or are in the presence of species complexes and hybrids [49,50]. In the present study, a low resolution of *COI* barcodes was highlighted during the analysis of products finally identified as belonging to species from *Merluccius* sp. and products finally not assigned at species level belonging to *Metapenaeus* sp. and *Heterocarpus* sp.

Limitations in species allocation have been described in the analysis of hake products (*Merluccius* sp.) for which the evaluation of different mitochondrial targets such as *cytb* or Control Region barcodes have been proposed in numerous studies [51]. In the present study, the use of *cytb*, in additional to *COI*, was conclusive for the product allocation to two species, *M. productus* and *M. gayi*. In fact, the similarity analysis on the *COI* target computed with BLASTn and K2P analysis computed with BOLD IDs had shown overlapping identity values within the threshold set for species–specific identification. The distance–based dendrogram produced on *cytb* target by including reference sequences belonging to 11 *Merluccius* sp. species and 11 sequences obtained in this study from products shows distinct and discrete species clustering and the allocation of the products sequences within the cluster *M. products* (F15, F16, F17, F46, F63, F66, F95) and *M. gayi* (F61, F79, F81, F82) clusters (Figure S1). Thus, in accordance with the multitarget approach proposed by Tinacci et al. [31], the introduction of an additional target offered a decisive improvement of the species disambiguation and the final allocation of the products at the species level.

With respect to *Metapenaeus* sp., although the DNA barcoding technique offers an efficient tool for species identification to ensure traceability and identity verification of crustacean products [52,53] in the present study, objective limitations were highlighted to the final species allocation within the genus even despite the use of the additional target *16S rRNA* or *PEPCK*. Similar considerations can be applied to the failure of species identification for a product (C10) identified as belonging to the genus *Heterocarpus* sp. Limits in molecular identification of penaeid shrimps were also highlighted by Rajkumar [54]. In this respect, several authors concurred in identifying the gene targets selected in the present study as valid for the identification of Penaeid shrimp species, nonetheless emphasizing the importance of selecting the bioinformatics method to be applied for an accurate definition of the relationships and clustering of the various species within genera clades [18,52,55].

#### 3.2.2. Reference Sequences’ Unreliability and Absence

Reference sequences’ unreliability and absence have been extensively debated and generally attributed to incorrect species–sequence associations or the submission of sequences belonging to not taxonomically validated specimens [56,57,58]. The presence of reference sequences of uncertain and debatable reliability was highlighted in the study during the post–sequencing analysis of *COI* target barcodes obtained from an MC product (MC32) and from five MG products (MG1, MG2, MG3, MG4 and MG5). In the first case, MC32 was finally assigned to the species *Notodarus sloanii* despite an initial overlapping top identity match of the MC32 *COI* sequence with the reference sequences of *Nototodarus sloanii* (100–99.84%), and 4 reference sequences (DQ373957–60) deposited by Carlini et al. [59] for *Illex argentinus* (100–99.84%). The four sequences were excluded by assuming the hypothesis of a potential error occurring during the morphological identification of the specimens. The assumption was strengthened by the results of a further cross–BLAST analysis of the sequences deposited by Carlini et al. [59] against the GenBank repository of all *I. argentinus COI* reference sequences from which the identity values lower than 85% were highlighted for each of the 4 investigated sequences. A similar assumption was pursued for the final allocation at the species level of the five MG products included in the study. In this respect, the post–sequencing analysis of the *COI* barcodes obtained from five MG products highlighted overlapping similarity scores within the ID score threshold (98%) with *R. venosa*, *R. bezoar* and three reference sequences deposited for *Turbo cornutus* (HM180929, HM180930 and HM180931) deposited by Kim at al. [60]. The three sequences were excluded following a cross–BLAST analysis against the GenBank repository of all *Turbo cornutus COI* sequences from which identity values lower than 78% were highlighted for each of them. Thus, the final allocation of the products to the species Rapana venosa was achieved through distance analysis conducted on the target *16S rRNA* (Table S3; Figure S2), which had been previously successfully applied in a comprehensive phylogeny study on Rapaninae, Muricidae taxonomical group in association with *COI*, *18S rRNA*, *12S rRNA* [61]. 

In the case of *Alepocephalus* sp., given the low resolution of the elective target for species–specific identification revealed during BLAST analysis, the analysis was not conclusive due to the lack of deposited reference sequences for the additional target gene provided by the operative protocol (*cytb*). This evidence underlined a major limitation of the DNA barcoding technique and the need to continuously update reference databases.

The need for continuous updating of the comprehensive DNA barcode reference libraries has been extensively emphasized in a study conducted by Weigand and colleagues [62] on the Barcode of Life Data Systems (BOLD) and NCBI GenBank databases. From this study a clear lack of homogeneity in the deposit of sequence records among taxonomic groups emerged among geographic regions. The authors also highlighted that the presence of a large proportion of species (up to 50%) in several taxonomic groups is only represented by private data with obvious implications on the actual possibility of their use. The authors, therefore, emphasize the need for a coordinated and systematic action of database improvement to close the information gap and to maximize phylogenetic representativeness, thereby yielding to the collection of reference barcodes of representative species from missing orders, families and genera.

### 3.3. Assessment of Products Labeling Compliance and Mislabeling Rate

#### 3.3.1. Labeling Compliance to Regulation (EU) No. 1379/2013

It is first appropriate to emphasize that according to Regulation (EU) No. 1379/2013 [16], the labeling obligations stated in Article 35 do not apply to processed products which, except for marinated and smoked products, do not fall within the scope of the provision. For other products, the application of the regulatory requirements is exclusively subject to the FBO’s will, although strongly advocated by the EU Parliament to promote informed consumer choice at the time of purchase [63]. Voluntary extensions of the regulation’s requirements for the labeling of out–of–scope products have been documented in numerous studies of species identification in variously processed seafood [64,65,66,67]. This considered the labels’ analysis confirmed for all the products within the scope of Regulation (EU) No. 1379/2013 [16] (N = 160), the proper application of Article 35, indicating commercial designation and species scientific name, an indication of production method, catching area, and thawing process. The only exception was represented by the fishing method, which was not declared in 6.8% of the products (11/160) (Table S1). This confirmed the substantial labeling compliance of products sold at large retail, as already highlighted by Tinacci et al. [9].

Interestingly, the analysis of the data also revealed the voluntary application of the Regulation requirements in the remaining products (N = 39), consisting of breaded precooked (N = 26) and canned (N = 13) products. In all these products the commercial designation and scientific name, origin and catching area was reported while the fishing method was only reported in 38.46% (15/39) of the products. Notwithstanding, since any information included on the label, whether mandatory or voluntarily introduced, is subject to transparency and authenticity obligations according to Regulation (EU) No. 1169/2011 (article 7) [15], all products under study were included in the calculation of the mislabeling rate overall and for the individual product categories (F, MC, MB, MG and C) discussed below. 

#### 3.3.2. Mislabeling Rate and Products Origin

In the comparison of the molecular results with the labeled scientific names, the presence of 22 substitutions out of 199 products, corresponding to an overall mislabeling rate of 11%, was found All substitution incidents are presented in Table 2.

The overall mislabeling rate appears to be lower than in the pilot study [22], which stood at around 17%. The highest percentage was found in WF (14%), followed by MB (12.5%) and MC (10%). The overall percentage and percentages per taxonomical class fall within the substitution range (4–14%) identified by Luque and Donlan [13] in a meta–analysis study conducted on scientific data and publications produced up to 2017. In particular, the WF mislabeling rate appears next to the percentage (12.9%) highlighted by Minoudi et al. [68] in a market survey conducted on the Greek market and mostly directed toward whitefish species. In this regard, we have to highlight that in 2015, the frequency and impact of fraud perpetrated on this category prompted the EU Commission to organize a coordinated control program across all member states to assess the extent of mislabeling in the fishery sector with a specific focus on the whitefish market [69].

Although not internationally harmonized, the definition of species substitution is broadly described as the intentional deception of a food product for economic gain or to conceal other illegal actions such as illegal, unreported and unregulated fishing achieved through the misrepresentation of food products or alteration of the associated documentation [11,70,71]. Conversely, in addition to fraudulent incidents, the existence of involuntary substitutions due to the lack of adequate training in morphological species identification of fishermen and operators at the first sale level should also be emphasized [72]. Both incidents result in damage to both consumers and food business operators and underline the fishery sector’s vulnerability and the need to acquire in–depth knowledge of seafood chain traceability systems and individual business practices [11,25].

Within WF substitution, incidents between Alaska pollock (*G. chalcogrammus*) and species belonging to *Merluccius* sp. of medium commercial value were frequently observed, followed by substitutions of Atlantic cod (*G. morhua*) with Saithe (*Pollachius virens*) and substitutions of hake species (*Merluccius productus* and *Merluccius hubbsi*) of medium commercial value replaced with Gadidae species of lower commercial value (*Micromesistius australis*). The substitutions observed in the study are all widely described and attributable to fraudulent incidents for economic gain [12,25,73,74]. Similar evidence within the Gadidae and Merluccidae families was found by Minoudi et al. [68], wherein they identified misidentification during fishing and plausible fraudulent actions perpetrated at the product distribution level as possible causes of substitution. The study of the natural geographical distribution of substitute and replaced species could provide useful elements to potentially determine the origin of the fraud, as most potentially perpetrated at the first sale or at an intermediate level during the products processing [75].

In the present study, the analysis of the labeled origins revealed a mostly non–Mediterranean supply mainly oriented to products of Atlantic or Pacific origin. Within WF, the fishing areas most frequently highlighted at purchase were Northeast/Northwest Pacific (FAO 61/67), corresponding to the distribution area of *G. chalcogrammus*. Less frequent but nonetheless relevant were Northeast/Northwest Atlantic (FAO 21/27) and Southeast Atlantic (FAO 41), corresponding to the distribution areas of Atlantic cod (*G. morhua*), Saithe (*P. virens*), Baird’s smooth–head (*Alepocephalus bairdii*) and a few hake species (*M. hubbsi*, *Macruronus* sp.) all appreciably represented in large–scale distribution. Product of Mediterranean origin and, in particular, Black Sea origin (FAO 37.4) was exclusively found in products labeled as *Merlangius merlangus* (whiting), a fish species belonging to the Gadidae family of local interest and of medium commercial value. 

Thus, from the observation of the results collected in Table 3, fraudulent substitution phenomena within WF possibly occurred both at fishing/first sale and during processing or packaging. In detail, fraudulent substitutions at the first–sale level are plausibly conceivable for replaced and substitute species that share the geographic area, as in the substitution of *G. chalcogrammus* with *M. productus* or *G. morhua* with *P. virens*. Conversely, fraudulent substitution phenomena occurring at a more advanced stage of the chain (processing, packaging and distribution) are speculated in cases where a geographically distant substitute species were highlighted, such as in the substitution of *G. chalcogrammus* with *M. hubbsi* and *M. productus* with *M. hubbsi* or *M. australis*. In terms of environmental sustainability, it is pertinent to emphasize that the perpetration of substitution between geographically distant products could also conceal the attempt to reallocate products belonging to illegal fishing [25].

Within MC, Humboldt squid (*D. gigas*) was verified as the dominant species on the market, bringing the Southwest and Southeast Pacific (FAO 81/87) into a prominent position among exporting areas. The species was also the most frequently substituted species in the pilot study [22] and in the studies concerning mislabeling of cephalopod products on the Chinese and EU retail market [76,77]. In fact, *D. gigas* has been thoroughly described as one of the elective substitute species, especially due to its high availability and low commercial value, which render the species an appealing candidate in the perpetration of deceptive frauds for economic gain [77,78]. 

In MB products, the analysis of products origin highlighted a market orientation towards imported products belonging to North Atlantic (FAO 27) or South Pacific (FAO 81/87), specifically represented by the Mediterranean mussel (*M. galloprovincialis*), Chilean mussel (*M. chilensis*) and New Zealand green–lipped mussel (*Perna canaliculus*). The only two substitutions encountered consisted of the replacement of *M. galloprovincialis* with *M. chilensis*. Similar substitutions are described in products imported from Chile and have been attributed to unintentional accidents related to the coexistence of the two species in fishing and aquaculture areas along Chilean coasts [21]. However, this observation does not apply to one of the two mislabeled products in the study (MB13), a canned marinated product, for which the clear North Atlantic (FAO 27) origin of the product was declared on the label and a fraudulent action is clearly hypothesized and collocable at the product processing stage. 

Lastly, in terms of C–class from the data collected at purchase, the market supply appeared directed to aquaculture products (*P. vannamei*) of Asian origin (Vietnam, Bangladesh, India) except for the sporadic presence of *P. borealis* of the Northwestern Atlantic origin and Metapenaeus sp./Penaeus sp. products from the Indian Ocean. Thus, with respect to the substitutions highlighted, given the considerations outlined above concerning the difficulties encountered in the morphological species identification [52] and given the high degree of overlap of the geographical distribution areas of the substituted and substituted species (Sealifebase.org), it is plausible to affirm the occurrence of involuntary substitution phenomena for these products. Nevertheless, this aspect further underlines the need to promote and improve the operator’s awareness and skills for species recognition, also implementing molecular identification systems that can be used at processing plants [79]. Improved traceability tools represent, in fact, an essential support for FBOs who, in any case, hold the responsibility of verifying the identity of their products for consumer protection in accordance with EU legislation [31]. In this regard, rapid identification methods have been promoted and developed in recent years for the most traded species, potentially applicable to self–monitoring by various operators, especially at the distribution level [80,81,82].

#### 3.3.3. Adherence of Labeled Designation to Official Designations Accepted in the National Territory

Table 3 presents the results of the comparison between the scientific names found in association with the products and the official list of accepted trade names in force during the sampling period [45], and the updated list [46].

The comparison of the labeled scientific names and those listed in the ministerial ordinance, including the accepted designations, clearly highlighted the ineffectiveness of the list in force at the time of sampling in describing the basket of species present on the market. This aspect is extensively investigated and described by Tinacci et al. [23] in a study aimed at assessing the validity and accuracy of the new official Bulgarian list of seafood trade names in compliance with EU requirements. The authors, in this regard, stated that there was a clear contradiction between the official records listed in the ordinance and the market needs. The authors particularly emphasized the ineffectiveness of the list in describing imported products that are widely available on the Bulgarian market and especially at the large retail level, which is even more evident in the newly promulgated list, in which some previously included scientific names of relevance, such as *G. morhua* or *Pollachius virens*, have disappeared. Therefore, an urgent need for further revision and expansion of the list of official denominations is advisable and pivotal to meet the obligation of periodic updating imposed by Regulation (EU) No. 1379/2013 (Article 37) [16] and to provide FBOs with an effective tool to guarantee consumer rights on informed choice.

In the revision of the list, as pointed out by Tinacci et al. [22], Tinacci et al. [23] and in the present study, all taxonomical classes should be considered since, albeit to a lesser extent, besides fish, both mollusks and crustaceans are variously represented on the national market. An example of exponential evolution and expansion of the list of official denominations in relation to market needs is presented by Tinacci et al. [83] for the Italian context. In a comparative retrospective analysis of the lists promulgated for the marketing of seafood products on the national territory from 2002 to 2017, the authors highlighted the market inputs at the origin of the evolution of the list, which were driven both by the demand of the average Italian consumer and by the ethnic groups and migrant populations permanently present on the national territory. 

Finally, regarding taxonomical validity, three invalid names, referring to an obsolete classification were highlighted (Table 3). It is, admittedly, true that updating scientific designations is an extremely challenging issue, given the continuous advancement in fish and seafood phylogeny research [83]. In this regard, therefore, in conjunction with the revision of the list in which, as highlighted, these names are not yet included, the promotion of a specific FBO training would be appropriate to monitor and encourage the replacement of obsolete names and update the labeling of new product batches. 

## 4. Conclusions

The overall mislabeling rate of 11% and the analysis of the substitution incidents that emerged in the study highlighted the need to promote the implementation of DNA–based monitoring systems oriented towards supplier selection to be applied amongst FBOs at various levels of the production chain (processing and retail). This could be reduce involuntary substitutions and prevent deceptive practices to protect both seafood supply chain and consumers’ rights. In this light, an integrated approach for species–specific polymorphisms analysis, through the association of different DNA analytical methods, may represent a useful and effective strategy for univocal seafood identification. In addition, this study confirms how the Bulgarian fish market, with reference to large–scale retail sales, still appears to be targeting a limited, albeit expanding, number of species. This aspect is stressed by the apparent inadequacy of the list of official names currently in force in the territory to describe the variety of products on sale. Therefore, as pointed out in a previously published study by the authors [23], a further update and expansion of the carnet of official commercial designations authorized in the territory are required. The data obtained from this survey could constitute inputs for implementing a monitoring plan promoted by governmental agencies in agreement with seafood stakeholders (wholesalers and sellers) for seafood authentication, contributing to the transparency of the seafood market at the national level.

## Figures and Tables

**Table 1:** Molecular targets selected in this study, primers pairs and amplification protocol applied. WF—whitefish; MB—bivalve mollusks; MC = cephalopod mollusks; MG—gastropod mollusks; C—crustaceans.

Target Length	Taxonomical Class	Reference	Primer Sequences	Amplification Program
*COI* (Elective) 655–658 bp	WF	Handy et al. [32]	FISH_COILBC TCAACYAATCAYAAAGATATYGGCAC FISH_COIHBC ACTTCYGGGTGRCCRAARAATCA	40 cycles: Denaturation 95 °C/30 s Annealing 55 °C/30 s Extension 72 °C/30 s
	MB	Mikkelsen et al. [33]	COIF–ALT ACAAATCAYAARGAYATYGG COIR–ALT TTCAGGRTGNCCRAARAAYCA	40 cycles: Denaturation 95 °C/30 s Annealing 47 °C/35 s Extension 72 °C/20 s
	MC MG C	Folmer et al. [34]	LCO1490 GGTCAACAAATCATAAAGATATTGG HC02198 TAAACTTCAGGGTGACCAAAAAATCA	40 cycles: Denaturation 95 °C/30 s Annealing 51 °C/30 s Extension 72 °C/30 s
*cytb* (additional) 1150 bp	WF	Sevilla et al. [35]	GLUFISH–F AACCACCGTTGTTATTCAACTACAA THR–Fish–R ACCTCCGATCTTCGGATTACAAGACC	40 cycles: Denaturation 95 °C/30 s Annealing 57 °C/30 s Extension 72 °C/45 s
*16S rRNA* (additional) ~550 bp	WF MB MC MG C	Palumbi [36]	16Sar_L CGCCTGTTTATCAAAAACAT 16Sbr_H CCGGTCTGAACTCAGATCACGT	40 cycles: Denaturation 95 °C/30 s Annealing 57 °C/15 s Extension 72 °C/15 s
*PEPCK* (additional) 595 bp	C	Tsang et al. [37]	PEPCK FOR2 GCAAGACCAACCTGGCCATGATGAC PEPCK REV3 CGGGYCTCCATGCTSAGCCARTG	40 cycles: Denaturation 95 °C/30 s Annealing 59 °C/30 s Extension 72 °C/35 s
*PAP* RFLP protocol	MB (*Mytilus* sp. only)	Giusti et al. [21]	Me15m–F GTATACAAACCTGTGAAGACAAGT Me16m–R TGTTGTCTTAATAGGTTTGTAAGATG *Aci–I* enzymatic digestion	40 cycles: Denaturation 95 °C/30 s Annealing 58 °C/30 s Extension 72 °C/30 s Incubation 37 °C/30 min Inactivation 65 °C/20 min

**Table 2:** List of mislabeled products. * Geographical distribution area verified from FAO databases (Sealife.org and FishBase.org).

Taxonomical Class Substitution Rate Percentage	Test Target	Code	Product Type, Description	Declared Scientific Name (Real Distribution Area) *	Molecular Identification (Real Distribution Area) *
Whitefish (WF) N = 100 Substitution rate: 14%	Barcoding *COI*	WF3	Unprocessed frozen, fillets	*Gadus morhua* (FAO 21–27)	*Pollachius virens* (FAO 21–27)
	Barcoding *COI*	WF6	Unprocessed frozen, fillets	*Gadus morhua* (FAO 21–27)	*Pollachius virens*
	Barcoding *COI, cytb*	WF15	Unprocessed frozen, w/o head and eviscerated	*Theragra chalcogramma* (FAO 61–67)	*Merluccius productus* (FAO 67)
	Barcoding *COI, cytb*	WF16	Unprocessed frozen, w/o head and eviscerated	*Theragra chalcogramma* (FAO 61–67)	*Merluccius productus* (FAO 67)
	Barcoding *COI, cytb*	WF17	Unprocessed frozen, w/o head and eviscerated	*Theragra chalcogramma* (FAO 61–67)	*Merluccius productus* (FAO 67)
	Barcoding *COI*	WF19	Unprocessed, frozen, fillets	*Theragra chalcogramma* (FAO 61–67)	*Merluccius hubbsi* (FAO 41)
	Barcoding *COI*	WF21	Unprocessed, frozen, fillets	*Theragra chalcogramma* (FAO 61–67)	*Merluccius hubbsi* (FAO 41)
	Barcoding *COI*	WF25	Unprocessed frozen, w/o head and eviscerated	*Macruronus magellanicus* (FAO 41–87)	*Merluccius hubbsi* (FAO 41)
	Barcoding *COI*	WF36	Unprocessed, frozen, fillets	*Gadus morhua* (FAO 21–27)	*Pollachius virens* (FAO 21–27)
	Barcoding *COI*	WF39	Unprocessed frozen, w/o head and eviscerated	*Merluccius hubbsi* (FAO 41)	*Micromesistius australis* (FAO 81)
	Barcoding *COI*	WF45	Unprocessed frozen, w/o head and eviscerated	*Merluccius productus* (FAO 67)	*Micromesistius australis* (FAO 81)
	Barcoding *COI*	WF47	Unprocessed, frozen, fillets	*Theragra chalcogramma* (FAO 61–67)	*Merluccius hubbsi* (FAO 41)
	Barcoding *COI, cytb*	WF79	Processed frozen, breaded fillets	*Theragra chalcogramma* (FAO 61–67)	*Merluccius gayi* (FAO 87)
Cephalopod Mollusks (MC) N = 40 Substitution rate: 10%	Barcoding *COI*	MC6	Unprocessed frozen, whole peeled and eviscerated	*Ommastrephes bartramii* (Cosmopolitan)	*Dosidicus gigas* (FAO 77–87)
	Barcoding *COI*	MC21	Unprocessed frozen, sliced	*Todarodes pacificus* (FAO 61)	*Dosidicus gigas* (FAO 77–87)
	Barcoding *COI*	MC22	Unprocessed frozen, whole peeled and eviscerated	*Ommastrephes bartramii* (Cosmopolitan)	*Dosidicus gigas* (FAO 77–87)
	Barcoding *COI*	MC40	Unprocessed frozen, sliced	*Nototodarus sloanii* (FAO 81)	*Todaropsis eblanae* (cosmopolitan, no FAO 77–87)
Bivalve Mollusks (MB) N = 16 Substitution rate: 12.5%	RFLP *PAP*	MB13	Processed canned	*Mytilus galloprovincialis* (FAO 27–37–41–87)	*Mytilus chilensis* (FAO 41–87)
	RFLP *PAP*	MB15	Processed canned	*Mytilus galloprovincialis* (FAO 27–37–41–87)	*Mytilus chilensis* (FAO 41–87)
Crustaceans (C) N = 38 Substitution rate: 7.9%	Barcoding *COI, PEPCK*	C10	Unprocessed frozen, w/o head peeled	*Solenocera Melantho* (FAO 61–71–81)	*Heterocarpus sp.* (FAO51–57–61–71)
	Barcoding *COI*	C31	Processed frozen, w/o head peeled precooked	*Metapenaeus sp* (FAO 51–57)	*Fenneropenaeus indicus* (FAO 51–57)
	Barcoding *COI*	C36	Unprocessed frozen, whole	*Solenocera Melantho* (FAO 61–71–81)	*Plesionika quasigrandis* (FAO 51–57)

**Table 3:** Comparison between the scientific names found on the labels, the scientific names included in Ministerial, the official list of designations in force during the sampling period and the updated list currently in force.

Species Labeled	Ordinance No. 4 of 13.01.2006	Ordinance No. 13 30.11.2021
*Theragra chalcogramma*	no	no
*Gadus morhua*	**yes**	no
*Merlangius merlangus euxinus*	**yes**	**yes**
*Pollachius virens*	**yes**	no
*Merluccius* sp.	no	no
*Merluccius hubbsi*	no	no
*Merluccius productus*	no	no
*Merluccius australis*	no	no
*Micromesistius australis*	no	no
*Macruronus magellanicus*	no	no
*Macruronus novaezelandiae*	no	no
*Macruronus australis*	no	no
*Alepocephalus bairdii*	no	no
*Litopenaeus vannamei*	no	no
*Penaeus monodon*	no	no
*Metapenaeus affinis*	no	no
*Metapenaeus* sp.	no	no
*Solenocera melantho*	**no**	no
*Pandalus borealis*	**yes**	no
*Nephrops norvegicus*	yes	no
*Dosidicus gigas*	no	no
*Ommastrephes bartramii*	no	no
*Illex argentinus*	no	no
*Todarodes pacificus*	no	no
*Nototodarus sloanii*	no	no
*Loligo gahi/patagonica*	no	no
*Octopus vulgaris*	no	no
*Sepia* sp.	no *(Sepia officinalis)*	no
*Mytilus chilensis*	**no**	**no**
*Mytilus galloprovincialis*	yes	yes
*Mytilus* sp.	no	no
*Perna canaliculus*	no	**no**
*Rapana venosa*	no	**yes**

## Data Availability

Data is included in the manuscript and available in supplementary tables attached to the text; further data is also available at the authors on request.

## Supplementary Materials

The following supporting information can be downloaded at: https://www.mdpi.com/article/10.3390/foods12051070/s1, Figure S1: Distance–based dendrogram inferred with Neighbor–joining method on Kimura 2 parameter model computed in MEGA 11 involving 67 nucleotide sequences which include a selection of 56 reference sequences belonging to species of the genus *Merluccius* sp and 11 sequences produced in the study (F15, F16, F17, F46, F61, F63, F66, F79, F81, F82 and F95) highlighted with the symbol (●). All positions containing gaps and missing data were eliminated. The percentage of replicate trees in which the associated taxa clustered together in the bootstrap test (1000 replicates) are shown above the branches, only bootstrap values higher than 70% are shown in the figure; Figure S2: Distance–based dendrogram inferred with Neighbor–joining method on Kimura 2 parameter model computed in MEGA 11 involving 37 nucleotide sequences which include 32 reference sequences belonging to R. bezoar, R. rapiformis, R. venosa, T. cornutus and five sequences produced in the study (MG 1, MG2, MG3, MG4 and MG5) highlighted with the symbol (●). The percentage of replicate trees in which the associated taxa clustered together in the bootstrap test (1000 replicates) are shown above the branches, only bootstrap values higher than 70% are shown in the figure; Table S1: Labeling information of the products collected in the study; Table S2: Results of molecular analysis of the products. Mislabeled products are highlighted in grey; Table S3: Estimates of interspecies divergence rate over sequence pairs between groups conducted with K2P model on a selection of 32 reference sequences deposited for *R. venosa* (N = 10); *R. bezoar* (N = 10); *R. rapiformis* (N = 7); *T. cornutus* (N = 5).
