# Supplementary material for: An Authentication Survey on Retail Seafood Products Sold on the Bulgarian Market Underlines the Need for Upgrading the Traceability System
Source: Foods. 2023 Mar 2;12(5):1070. doi: 10.3390/foods12051070 (PMC10000581; doi:10.3390/foods12051070)
Supplement: Supplementary file 1 [file foods-12-01070-s001.zip › foods-2211350-supplementary.pdf]

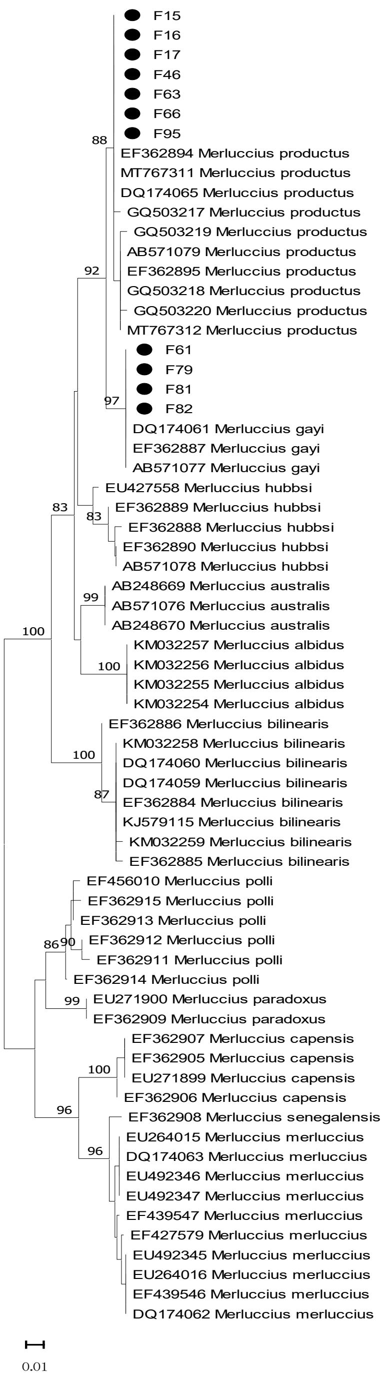

Figure S1. Distance based dendrogram inferred with Neighbour-Joining method on Kimura 2 parameter model computed in MEGA 11 involving 67 nucleotide sequences which include a selection of 56 reference sequences belonging to species of the genus *Merluccius* sp and 11 sequences produced in the study (F15, F16, F17, F46, F61, F63, F66, F79, F81, F82, F95) highlighted with the symbol (•). All positions containing gaps and missing data were eliminated. The percentage of replicate trees in which the associated taxa clustered together in the bootstrap test (1000 replicates) are shown above the branches, only bootstrap values higher than 70% are shown in the figure.

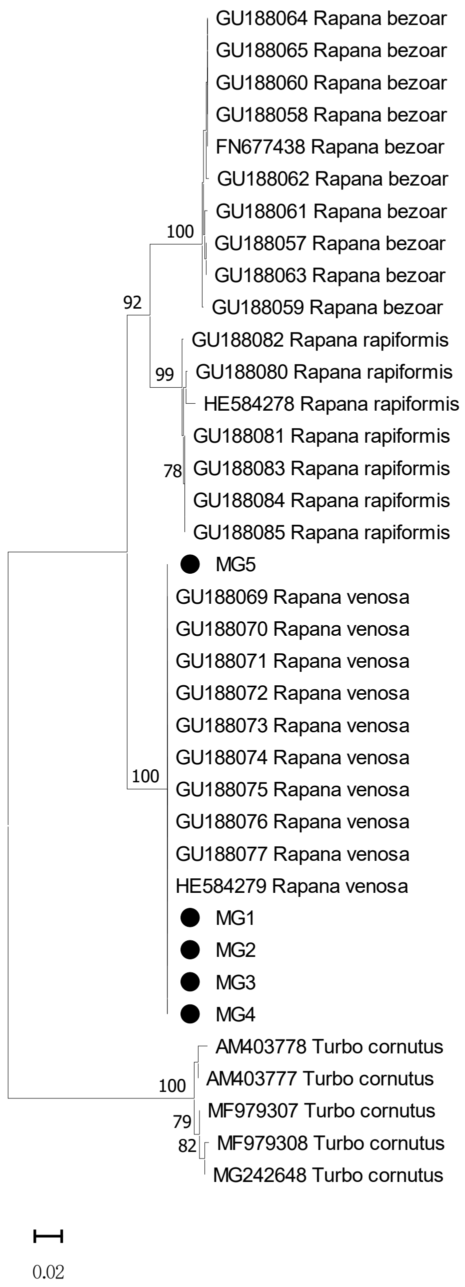

Figure S2 Distance based dendrogram inferred with Neighbour-Joining method on Kimura 2 parameter model computed in MEGA 11 involving 37 nucleotide sequences which include 32 reference sequences belonging *to R. bezoar, R. rapiformis, R. venosa, T. cornutus* and 5 sequences produced in the study (MG 1, MG2, MG3, MG4, MG5) highlighted with the symbol (●). The percentage of replicate trees in which the associated taxa clustered together in the bootstrap test (1000 replicates) are shown above the branches, only bootstrap values higher than 70% are shown in the figure.

Sample code	Product type	Description	Purchasing point	Label information					
				Commercial name		Scientific name	Geographical origin	Catching / farming area	Category of fishing gear
				Bulgarian	English				
WF1	Unprocessed Frozen	W/o head eviscerated	Stara Zagora	Меджид	Whiting	*Merlangius merlangus euxinus*	Black Sea	FAO 37.4	Trawling net
WF2	Unprocessed Frozen	Fillets	Stara Zagora	Хек	Hake	*Theragra chalcogramma*	Pacific Ocean	FAO 61	Trawling net
WF3	Unprocessed Chilled	Fillets	Stara Zagora	Треска	Cod	*Gadus morhua*	Atlantic Ocean	FAO 27	Trawling net
WF4	Unprocessed Frozen	W/o head eviscerated	Stara Zagora	Мерлуза	Merluccius	*Merluccius hubbsi*	Spain	FAO 41	Trawling net
WF5	Unprocessed Frozen	Fillets	Stara Zagora	Треска	Cod	*Theragra chalcogramma*	China	FAO 61	Trawling net
WF6	Unprocessed Chilled	Fillets	Stara Zagora	Треска	Cod	*Gadus morhua*	Iceland	FAO 27.5	Trawling net
WF7	Unprocessed Frozen	Fillets – mix of fish	Stara Zagora	Атлантическа треска	Atlantic cod	*Gadus morhua*	Atlantic Ocean	FAO 27 - Norwegian Sea or FAO 21 - Atlantic Ocean	Trawling net
WF8	Unprocessed Frozen	Fillets	Stara Zagora	Минтай	Alaska pollock	*Theragra chalcogramma*	Poland	FAO 61 or FAO 67	Trawling net
WF9	Processed Frozen	Breaded precooked Fillets	Stara Zagora	Морска треска	Alaska pollock	*Theragra chalcogramma*	Okhotsk sea and Bering sea	FAO 61 and FAO 67	-
WF10	Unprocessed Frozen	W/o head eviscerated	Stara Zagora	Мерлуза	Merluccius	*Macruronus magellanicus*	Falkland Islands	FAO 41	Trawling net
WF11	Unprocessed Frozen	W/o head eviscerated	Stara Zagora	Хек	Hake	*Merluccius hubbsi*	Spain	FAO 41	Trawling net

WF12	Unprocessed Frozen	W/o head eviscerated	Stara Zagora	Хек	Hake	*Theragra chalcogramma*	China	FAO 61	Trawling net
WF13	Unprocessed Frozen	Fillets	Stara Zagora	Треска	Cod	*Theragra chalcogramma*	China	FAO 61	Trawling net
WF14	Unprocessed Frozen	Fillets	Stara Zagora	Хек	Hake	*Merluccius hubbsi*	Atlantic Ocean	FAO 41	Trawling net
WF15	Unprocessed Frozen	W/o head eviscerated	Stara Zagora	Хек	Hake	*Theragra chalcogramma*	China	FAO 61	Trawling net
WF16	Unprocessed Frozen	W/o head eviscerated	Stara Zagora	Хек	Hake	*Theragra chalcogramma*	China	FAO 61	Trawling net
WF17	Unprocessed Frozen	W/o head eviscerated	Stara Zagora	Хек	Hake	*Theragra chalcogramma*	Spain	FAO 27	Trawling net
WF18	Unprocessed Frozen	Fillets	Stara Zagora	Хек	Hake	*Theragra chalcogramma*	China	FAO 61	Trawling net
WF19	Unprocessed Frozen	Fillets	Stara Zagora	Хек	Hake	*Theragra chalcogramma*	Spain	FAO 27	Trawling net
WF20	Unprocessed Frozen	Fillets	Stara Zagora	Хек	Hake	*Theragra chalcogramma*	Atlantic Ocean	FAO 41	Trawling net
WF21	Unprocessed Frozen	Fillets	Stara Zagora	Хек	Hake	*Theragra chalcogramma*	China	FAO 61	Trawling net
WF22	Unprocessed Frozen	Fillets	Stara Zagora	Треска	Cod	*Alepocephalus bairdii*	Spain	FAO 27	Trawling net
WF23	Unprocessed Frozen	W/o head eviscerated	Stara Zagora	Хек	Hake	*Theragra chalcogramma*	China	FAO 61	Trawling net
WF24	Unprocessed Frozen	Fillets	Stara Zagora	Треска	Cod	*Theragra chalcogramma*	China	FAO 61	Trawling net

WF25	Unprocessed Frozen	W/o head eviscerated	Stara Zagora	Мерлуза	Hake	*Macruronus magellanicus*	Spain	FAO 41	Trawling net
WF26	Unprocessed Frozen	Fillets –	Stara Zagora	Сайда	Saithe	*Pollachius virens*	Atlantic Ocean	FAO 27 – Iceland, Faroe Islands	Trawling net
WF27	Unprocessed Frozen	Fillets	Stara Zagora	Атлантическа треска	Atlantic cod	*Gadus morhua*	Atlantic Ocean	FAO 27 – Norwegian Sea or FAO 21 – Atlantic Ocean	Trawling net
WF28	Unprocessed Frozen	Fillets	Stara Zagora	Треска	Cod	*Theragra chalcogramma*	Pacific ocean	FAO 61 or FAO 67	Trawling net
WF29	Unprocessed Frozen	Fillets	Stara Zagora	Сайда	Saithe	*Pollachius virens*	Atlantic Ocean	FAO 27 – Iceland, Faroe Islands	Trawling net
WF30	Unprocessed Frozen	Fillets	Stara Zagora	Атлантическа треска	Atlantic cod	*Gadus morhua*	Atlantic Ocean	FAO 27 – Norwegian Sea or FAO 21 – Atlantic Ocean	Trawling net
WF31	Unprocessed Frozen	W/o head eviscerated	Stara Zagora	Мерлуза	Hake	*Macruronus novaezelandiae*	Folkland islands	FAO 41	Trawling net
WF32	Unprocessed Frozen	W/o head eviscerated	Stara Zagora	Мерлуза	Hake	*Macruronus novaezelandiae*	Spain	FAO 41	Trawling net
WF33	Unprocessed Frozen	W/o head eviscerated	Stara Zagora	Хек	Hake	*Theragra chalcogramma*	China	FAO 61	Trawling net
WF34	Unprocessed Frozen	Fillets	Stara Zagora	Треска	Cod	*Theragra chalcogramma*	China	FAO 61	Trawling net
WF35	Unprocessed Frozen	Fillets	Stara Zagora	Хек	Hake	*Alepocephalus bairdii*	Spain	FAO 27	Trawling net

WF36	Unprocessed Frozen	Fillets	Stara Zagora	Треска	Cod	*Gadus morhua*	Iceland	FAO 27.5	Trawling net
WF37	Unprocessed Frozen	W/o head eviscerated	Stara Zagora	Хек	Hake	*Theragra chalcogramma*	China	FAO 61	Trawling net
WF38	Unprocessed Frozen	W/o head eviscerated	Stara Zagora	Хек	Hake	*Theragra chalcogramma*	China	FAO 61	Trawling net
WF39	Unprocessed Frozen	W/o head eviscerated	Stara Zagora	Хек	Hake	*Merluccius hubbsi*	New Zealand	FAO 41	Trawling net
WF40	Unprocessed Frozen	W/o head eviscerated	Stara Zagora	Хек	Hake	*Merluccius hubbsi*	Spain	FAO 41	Trawling net
WF41	Unprocessed Frozen	Fillets	Stara Zagora	Треска	Cod	*Theragra chalcogramma*	Pacific Ocean	FAO 61 or FAO 67	Trawling net
WF42	Unprocessed Frozen	W/o head eviscerated	Stara Zagora	Хек	Hake	*Merluccius hubbsi*	Atlantic Ocean	FAO 41	Trawling net
WF43	Unprocessed Frozen	W/o head eviscerated	Stara Zagora	Меджит	Whiting	*M. merlangus euxinus*	Bulgaria/Black Sea	FAO 37.4	Trawling net
WF44	Unprocessed Frozen	W/o head eviscerated	Stara Zagora	Хек	Hake	*Theragra chalcogramma*	China	FAO 61	Trawling net
WF45	Unprocessed Frozen	W/o head eviscerated	Varna	Хек-Аляска	Hake-Alaska	*Merluccius productus*	New Zealand	FAO 67	Trawling net
WF46	Unprocessed Frozen	Fillets	Varna	Хек	Hake	*Merluccius productus*	China	FAO 67	Trawling net
WF47	Unprocessed Frozen	Fillets	Shumen	Хек	Hake	*Theragra chalcogramma*	China	FAO 61	Trawling net
WF48	Unprocessed Frozen	Fillets	Shumen	Бяла риба	Whitefish	*Theragra chalcogramma*	China	FAO 61	Trawling net

WF49	Unprocessed Frozen	Fillets	Shumen	Треска	Cod	*Theragra chalcogramma*	China	FAO 61	Trawling net
WF50	Unprocessed Frozen	Fillets	Shumen	Хек	Hake	*Merluccius hubbsi*	Poland	FAO 41	Trawling net
WF51	Unprocessed Frozen	Fillets	Shumen	Треска	Cod	*Alepocephalus bairdii*	Spain	FAO 27	Trawling net
WF52	Unprocessed Frozen	Fillets	Shumen	Треска	Cod	*Theragra chalcogramma*	China	FAO 61	Trawling net
WF53	Unprocessed Frozen	Fillets	Shumen	Треска	Cod	*Theragra chalcogramma*	France/ Pacific Ocean	FAO 61 or 67	Trawling net
WF54	Unprocessed Frozen	Fillets	Shumen	Атлантическа треска	Atlantic cod	*Gadus morhua*	Atlantic Ocean	FAO 27 – Norwegian Sea or FAO 21 – Atlantic Ocean	Trawling net
WF55	Unprocessed Frozen	Fillets	Shumen	Сайда	Saithe	*Pollachius virens*	Atlantic Ocean	FAO 27 – Iceland, Faroe Islands	Trawling net
WF56	Processed Frozen	Fish fingers	Shumen	Минтай	Alaska pollock	*Theragra chalcogramma*	Pacific Ocean	FAO 61 or 67	Trawling net
WF57	Processed Frozen	Fish fingers	Shumen	Минтай	Alaska pollock	*Theragra chalcogramma*	Pacific Ocean	FAO 61 or 67	Trawling net
WF58	Processed Frozen	Fish fingers	Shumen	Минтай	Alaska pollock	*Theragra chalcogramma*	Pacific Ocean	FAO 61 or 67	Trawling net
WF59	Processed Frozen	Fish fingers	Varna	Минтай	Alaska pollock	*Theragra chalcogramma*	Pacific Ocean	FAO 61 or 67	Trawling net
WF60	Processed Frozen	Precooked fillets with vegetables	Varna	Минтай	Alaska pollock	*Theragra chalcogramma*	Pacific Ocean	FAO 61 or 67	Trawling net
WF61	Processed Frozen	Fish fingers	Dobrich	Хек	Hake	*Merluccius* sp.	Estonia/ Pacific Ocean	FAO 67 or 87	Trawling net
WF62	Processed Frozen	Breaded fish fillets	Dobrich	Минтай	Alaska pollock	*Theragra chalcogramma*	Poland/ Pacific Ocean	FAO 61 or 67	Trawling net

WF63	Processed Frozen	Fish fingers	Dobrich	Хек	Hake	*Merluccius* sp.	Pacific Ocean / Atlantic Ocean	FAO 87 or 41	Trawling net
WF64	Unprocessed Frozen	Fillets	Dobrich	Мерлуза	Hake	*Merluccius hubbsi*	Falkland Islands	FAO 41	Trawling net
WF65	Unprocessed Frozen	W/o head eviscerated	Stara Zagora	Хоки	Hoky	*Macruronus novaezelandiae*	Spain/ Atlantic Ocean	FAO 41	Trawling net
WF66	Unprocessed Frozen	Fillets	Stara Zagora	Хек	Hake	*Merluccius productus*	China	FAO 67	Trawling net
WF67	Unprocessed Frozen	Fillets	Stara Zagora	Треска	Cod	*Alepocephalus bairdii*	Spain	FAO 27	Trawling net
WF68	Unprocessed Frozen	Fillets	Shumen	Хек	Hake	*Alepocephalus bairdii*	Spain	FAO 27	Trawling net
WF69	Unprocessed Frozen	Fillets	Shumen	Треска	Cod	*Theragra chalcogramma*	China	FAO 61	Trawling net
WF70	Unprocessed Frozen	Fillets	Shumen	Мерлуза	Hake	*Merluccius hubbsi*	Falkland Islands	FAO 41	Trawling net
WF71	Unprocessed Frozen	W/o head eviscerated	Shumen	Хоки	Hoky	*Macruronus magellanicus*	Spain/ Atlantic ocean	FAO 41	Trawling net
WF72	Unprocessed Frozen	Fillets	Stara Zagora	Треска	Cod	*Alepocephalus bairdii*	Spain	FAO 27	Bottom trawls
WF73	Unprocessed Frozen	W/o head eviscerated	Stara Zagora	Хек	Hake	*Micromesistius australis*	Spain	FAO 41	Pelagic trawls
WF74	Unprocessed Frozen	Fillets	Stara Zagora	Минтай	Alaska pollock	*Theragra chalcogramma*	Pacific Ocean	FAO 61	Trawling net
WF75	Unprocessed Frozen	Fillets	Stara Zagora	Бяла риба	White fish	*Merluccius hubbsi*	Spain	FAO 41	Trawling net

WF76	Unprocessed Frozen	W/o head eviscerated	Stara Zagora	Хоки	Hoki	*Macruronus magellanicus*	Spain	FAO 41	-
WF77	Unprocessed Frozen	Fillets	Stara Zagora	Минтай	Alaska pollock	*Theragra chalcogramma*	Pacific Ocean	FAO 61 and 67	Trawling net
WF78	Processed Frozen	Breaded fish fingers	Stara Zagora	Минтай	Alaska pollock	*Theragra chalcogramma*	Pacific Ocean	FAO 61 and 67	-
WF79	Processed Frozen	Breaded fillets	Stara Zagora	Минтай	Alaska pollock	*Theragra chalcogramma*	Pacific Ocean	FAO 61 and 67	Trawling net
WF80	Unprocessed Frozen	Fillets	Stara Zagora	Морска треска	Alaska pollock	*Theragra chalcogramma*	Sea of Okhotsk and Bering Sea	FAO 61 and 67	-
WF81	Unprocessed Frozen	Breaded fillets	Stara Zagora	Хек	Hake	*Merluccius sp.*	-	FAO 87, 67, 41	-
WF82	Processed Frozen	Breaded fillets	Stara Zagora	Хек	Hake	*Merluccius sp.*	Pacific Ocean	FAO 87,67	-
WF83	Processed Frozen	Breaded fillets	Stara Zagora	Морска треска	Alaska pollock	*Theragra chalcogramma*	Sea of Okhotsk and Bering Sea	FAO 61 and 67	Trawling net
WF84	Processed Frozen	Breaded fillets	Stara Zagora	Минтай	Alaska pollock	*Theragra chalcogramma*	Sea of Okhotsk and Bering Sea	FAO 61 and 67	Trawling net
WF85	Unprocessed Frozen	W/o head eviscerated	Stara Zagora	Меджит	Whiting	*Merlangius merlangus euxinus*	Bulgaria/Black Sea	FAO 37,4	Trawling net
WF86	Unprocessed Frozen	W/o head eviscerated	Stara Zagora	Аржентински хек	Argentine hake	*Merluccius hubbsi*	Spain/Atlantic Ocean	FAO 41	Trawling net
WF87	Unprocessed Frozen	Fillets	Shumen	Аржентински хек	Argentine hake	*Merluccius hubbsi*	Argentina/Atlantic Ocean	FAO 41	Trawling net
WF88	Unprocessed Frozen	Fillets	Shumen	Минтай	Hake	*Theragra chalcogramma*	Pacific ocean	FAO 61 or 67	Trawling net
WF89	Processed Frozen	Fish fingers	Shumen	Новозеландски макруронус	Grenadier albastru	*Macruronus novaezelandiae*	Pacific ocean	FAO 81	Trawling net
WF90	Unprocessed Frozen	W/o head eviscerated	Varna	Хек	Hake	*Merluccius hubbsi*	Atlantic Ocean	FAO 41	Trawling net

WF91	Unprocessed Frozen	W/o head eviscerated	Varna	Хек	Hake	*Merluccius australis*	Spain	FAO 27	Trawling net
WF92	Unprocessed Frozen	Fillets	Varna	Хек	Hake	*Alepocephalus bairdii*	Spain	FAO 67	Trawling net
WF93	Processed Frozen	Fish fingers	Dobrich	Минтай	Hake	*Theragra chalcogramma*	Pacific ocean	FAO 61 or 67	Trawling net
WF94	Unprocessed Frozen	W/o head eviscerated	Dobrich	Хоки	Hoky	*Macrruronus magellanicus*	Spain/ Atlantic ocean	FAO 41	Trawling net
WF95	Unprocessed Frozen	Fillets	Shumen	Хек	Hake	*Merluccius productus*		FAO 67	Trawling net
WF96	Unprocessed Frozen	Fillets	Shumen	Треска	Cod	*Theragra chalcogramma*	China	FAO 61	Trawling net
WF97	Unprocessed Frozen	Fillets	Shumen	Хек	Hake	*Alepocephalus bairdii*	Spain	FAO 27	Trawling net
WF98	Unprocessed Frozen	Fillets	Stara Zagora	Треска	Alaska pollock	*Theragra chalcogramma*	Pacific Ocean	FAO 61 or 67	Trawling net
WF99	Unprocessed Frozen	W/o head eviscerated	Stara Zagora	Хек	Hake	*Theragra chalcogramma*	China	FAO 61	Trawling net
WF100	Unprocessed Frozen	W/o head eviscerated	Stara Zagora	Мерлуза	Hake	*Micromesistius australis*	Spain	FAO 41	Trawling net
MC1	Processed Marinated, canned	Tentacle	Stara Zagora	Калмари	-	*Dosidicus gigas*	-	FAO 67	-
MC2	Processed Ready to eat	Slices	Stara Zagora	Сепия	-	*Sepia spp.*	Italy	FAO 37	-
MC3	Processed Ready to eat	Slices	Stara Zagora	Октоподи	-	*Octopus vulgaris*	Italy	FAO 37	-
MC4	Unprocessed Frozen	Tentacles	Stara Zagora	Калмари	-	*Dosidicus gigas*	Spain	FAO 87	Hand lines and pole lines (mechanised)
MC5	Unprocessed Frozen	Cleaned	Stara Zagora	Калмари	Jumbo squid	*Dosidicus gigas*	Pacific Ocean	FAO 87	Trawl
MC6	Unprocessed	Cleaned tubes	Stara Zagora	Калмари	Squid	*Ommastrephes bartrami*	Pacific Ocean	FAO 61	Hand lines and

	Frozen								pole lines (mechanised)
MC7	Processed Frozen	Breaded slices	Varna	Калмари	-	*Dosidicus gigas*	China	FAO 61	-
MC8	Processed Frozen	Breaded slices	Shumen	Калмари	Jumbo squid	*Dosidicus gigas*	China	FAO 61	-
MC9	Processed Frozen	Breaded slices	Shumen	Калмари	-	*Dosidicus gigas*	Pacific Ocean	FAO 77 or FAO 87	Trawl
MC10	Processed Frozen	Breaded slices	Stara Zagora	Калмари	Jumbo squid	*Dosidicus gigas*	China	FAO 61	-
MC11	Processed Frozen	Breaded slices	Shumen	Калмари	Jumbo squid	*Dosidicus gigas*	China	FAO 61	-
MC12	Unprocessed Frozen	Tentacles	Stara Zagora	Октопод	-	*Octopus vulgaris*	Atlantic ocean	FAO 34	Pots and traps
MC13	Unprocessed Frozen	Cleaned	Varna	Октопод	-	*Octopus vulgaris*	Atlantic ocean	FAO 34	Pots and traps
MC14	Unprocessed Frozen	Tubes	Shumen	Калмари	-	*Todarodes pacificus*	China	FAO 61	Trawl
MC15	Processed Frozen	Breaded pieces	Varna	Калмари	-	*Loligo patagonica*	Atlantic Ocean	FAO 41	Trawl
MC16	Unprocessed Frozen	Eviscerated without skin	Varna	Калмари	-	*Loligo gahi*	Atlantic Ocean	FAO 41	Trawl
MC17	Unprocessed Frozen	Tentacles	Varna	Калмари	-	*Dosidicus gigas*	Spain	FAO 87	Hand lines and pole lines (mechanised)
MC18	Unprocessed Frozen	Sliced and tentacles	Varna	Калмари	-	*Loligo patagonica*/ *Loligo gahi*	Atlantic Ocean	FAO 41	Trawl
MC19	Processed Frozen	Breaded	Dobrich	Калмари	-	*Dosidicus gigas*	-	FAO 61	-
MC20	Processed Frozen	Breaded	Shumen	Калмари	-	*Illex argentinus*	-	-	-

MC21	Unprocessed Frozen	Tubes	Stara Zagora	Калмари	-	*Todarodes pacificus*	Chine	FAO 61	Trawl
MC22	Unprocessed Frozen	Cleaned tubes	Varna	Калмари	Squid	*Ommastrephes bartrami*	Pacific Ocean	FAO 61	Hand lines
MC23	Processed Marinated	Tentacles	Stara Zagora	Калмари	-	*Dosidicus gigas*	-	FAO 87	-
MC24	Processed Grilled	Tentacles	Stara Zagora	Калмари	-	*Dosidicus gigas*	-	FAO 87	-
MC25	Processed canned in sauce	Pieces	Stara Zagora	Калмари	Squid	*Dosidicus gigas*	-	FAO 87	-
MC26	Processed canned in sauce	Pieces	Stara Zagora	Калмари	Squid	*Dosidicus gigas*	-	FAO 87	-
MC27	Processed canned in oil	Pieces	Stara Zagora	Калмари	Squid	*Dosidicus gigas*	-	FAO 61	-
MC28	Processed canned in oil	Pieces	Stara Zagora	Калмари	Squid	*Dosidicus gigas*	-	FAO 87	-
MC29	Processed precooked Frozen	Squid rings	Stara Zagora	Калмари	-	*Dosidicus gigas*	China	FAO 87	Hooks and lines
MC30	Processed precooked Frozen	Squid rings	Stara Zagora	Калмари	-	*Dosidicus gigas*	China	FAO 87	Hooks and lines
MC31	Processed precooked Frozen	squid rings and tentacles	Stara Zagora	Калмари	-	*Loligo patagonica/ Loligo gahi*	Marocco	FAO 41	Trawl
MC32	Unprocessed Frozen	squid rings and tentacles	Stara Zagora	Калмари	-	*Nototodarus sloanii*	New Zealand	FAO 81	Trawl
MC33	Unprocessed Frozen	Whole	Stara Zagora	Калмари	-	*Dosidicus gigas*	China	FAO 81	-
MC34	Unprocessed Frozen	squid tentacles	Stara Zagora	Калмари	-	*Dosidicus gigas*	Peru	FAO 81	Hooks and lines
MC35	Unprocessed	squid tentacles	Stara Zagora	Калмари	-	*Dosidicus gigas*	Spain	FAO 81	-

	Frozen								
MC36	Unprocessed Frozen	squid tentacles	Stara Zagora	Калмари	-	*Dosidicus gigas*	-	FAO 81	Hooks and lines
MC37	Unprocessed Frozen	squid tentacles	Varna	Калмари	-	*Dosidicus gigas*	Peru	FAO 81	Hooks and lines
MC38	Unprocessed Frozen	Whole	Varna	Калмари	-	*Loligo patagonica/ Loligo gahi*	Morocco	FAO 41	Trawl
MC39	Unprocessed Frozen	Pieces	Varna	Калмари	-	*Dosidicus gigas*	-	FAO 61	-
MC40	Unprocessed Frozen	Pieces	Varna	Калмари	-	*Nototodarus sloanii*	New Zealand	FAO 81	-
MB1	Processed Canned marinated	Edible part	Stara Zagora	Миди	Mussels	*Mytilus chilensis*	-	FAO 87	Manual
MB2	Processed Canned marinated	Edible part	Stara Zagora	Миди	Mussels	*Mytilus chilensis*	Chile	FAO 87	-
MB3	Processed canned	Marinated	Stara Zagora	Миди	Mussels	*Mytilus galloprovincialis*	Atlantic Ocean	FAO 27	-
MB4	Processed Canned	Edible part	Shumen	Миди	Mussels	*Mytilus chilensis*	Chile	FAO 87	Aquaculture
MB5	Unprocessed Frozen	Edible part	Stara Zagora	Зеленоуста мида	-	*Perna canaliculus*	New Zealand	FAO 81	-
MB6	Unprocessed Frozen	Edible part	Stara Zagora	Миди	Mussels	*Mytilus chilensis*	Chile	FAO 87	Aquaculture
MB7	Unprocessed Frozen	Edible part	Dobrich	Миди	Mussels	*Mytilus chilensis*	Chile	FAO 87	Aquaculture
MB8	Unprocessed Frozen	Edible part	Stara Zagora	Миди	Mussels	*Mytilus galloprovincialis*	Spain	FAO 27	Trawl

MB9	Unprocessed Frozen	Edible part	Shumen	Сини миди	-	*Mytilus sp.*	Chile	-	Aquaculture
MB10	Unprocessed Frozen	Edible part	Dobrich	Зеленоуста мида	-	*Perna canaliculus*	New Zealand	FAO 81	-
MB11	Processed frozen	Edible part	Stara Zagora	Миди	Mussels	*Mytilus chilensis*	Chile	FAO 87	Aquaculture
MB12	Marinated canned	Edible part	Shumen	Миди	Mussels	*Mytilus chilensis*	Chile	-	-
MB13	Processed Marinated, canned	Edible part	Stara Zagora	Миди	-	*Mytilus galloprovincialis*	Atlantic Ocean	FAO 27	-
MB14	Frozen	Edible part	Stara Zagora	-	Mussels	*Perna canaliculus*	New Zealand	-	Aquaculture
MB15	Processed Marinated, canned	Edible part	Stara Zagora	Миди	Mussels	*Mytilus galloprovincialis*	Spain	FAO 27	-
MB16	Processed Marinated, canned	Edible part	Stara Zagora	Миди	Mussels	*Mytilus galloprovincialis*	Spain	FAO 27	-
MG1	Processed canned Marinated	Edible part	Stara Zagora	Рапани	-	*Rapana venosa*	-	FAO 37.4.2	Manual
MG2	Processed canned Marinated	Edible part	Stara Zagora	Рапани	-	*Rapana venosa*	-	FAO 37.4.2	Manual
MG3	Processed canned Marinated	Edible part	Shumen	Рапани	-	*Rapana venosa*	-	FAO 37.4.2	Manual
MG4	Unprocessed Frozen	Pieces	Varna	Маринован и рапани	-	*Rapana venosa*	-	FAO 37.4.2	Manual
MG5	Unprocessed Frozen	Pieces	Varna	Маринован и рапани	-	*Rapana venosa*	-	FAO 37.4.2	Manual
C1	Unprocessed Frozen	Whole	Stara Zagora	Гренландск и скариди	-	*Pandalus borealis*	Greenland	FAO 21	Trawl

C2	Unprocessed Frozen	Peeled without head	Stara Zagora	Скариди „Ванамей“	Vannamei shrimp	*Litopenaeus vannamei*	Vietnam	Aquaculture	NA
C3	Unprocessed Frozen	Peeled	Stara Zagora	Скариди „Ванамей“	Vannamei shrimp	*Litopenaeus vannamei*	Vietnam	Aquaculture	NA
C4	Unprocessed Frozen	Peeled without head	Stara Zagora	Скариди „Ванамей“	Vannamei shrimp	*Litopenaeus vannamei*	Vietnam	Aquaculture	NA
C5	Unprocessed Frozen	Peeled without head	Stara Zagora	Скариди	Black Tiger Shrimp	*Penaeus monodon*	Bangladesh	Aquaculture	NA
C6	Unprocessed Frozen	Peeled without head	Stara Zagora	Скариди	Seawater Prawns	*Litopenaeus vannamei*	India	Aquaculture	NA
C7	Unprocessed Frozen	Peeled	Stara Zagora	Скариди	Seawater Prawns	*Litopenaeus vannamei*	Vietnam	Aquaculture	NA
C8	Unprocessed Frozen	Peeled	Stara Zagora	Скариди „Ванамей“	Whiteleg shrimp	*Litopenaeus vannamei*	India	Aquaculture	NA
C9	Unprocessed Frozen	Peeled	Stara Zagora	Скариди – белени	-	*Metapenaeus affinis*	India	FAO 51	Trawl
C10	Unprocessed Frozen	Peeled	Varna	Коктейлни скариди	-	*Solenocera Melantho*	China	FAO 61	Trawl
C11	Unprocessed Frozen	Whole	Varna	Скариди	Whiteleg shrimp	*Litopenaeus vannamei*	Ecuador	Aquaculture	NA
C12	Unprocessed Frozen	Whole	Varna	Норвежки скариди	-	*Nephrops Norvegicus*	Holland	FAO 27/IV B	Trawl
C13	Processed preooked frozen	Breaded	Varna	Панирани скариди	Breaded shrimps	*Litopenaeus vannamei*	Vietnam	Aquaculture	NA
C14	Unprocessed Thawed	Whole	Varna	Скариди	Seawater Prawns	*Penaeus monodon*	Vietnam	Aquaculture	NA
C15	Unprocessed Thawed	Whole	Varna	Скариди	Seawater Prawns	*Litopenaeus vannamei*	Vietnam	Aquaculture	NA

C16	Unprocessed Frozen	Whole	Varna	Скариди – цели	Black Tiger Shrimp	*Penaeus monodon*	Bangladesh	Aquaculture	NA
C17	Unprocessed Frozen	Peeled without head	Varna	Скариди „Ванамей“	Vannamei shrimp	*Litopenaeus vannamei*	Vietnam	Aquaculture	NA
C18	Unprocessed Frozen	Cooked	Shumen	Скариди – бланширани	Seawater Prawns	*Litopenaeus vannamei*	Vietnam	Aquaculture	NA
C19	Unprocessed Thawed	Whole	Shumen	Скариди „Ванамей“	-	*Penaeus vannamei*	Columbia	Aquaculture	NA
C20	Unprocessed Thawed	Whole	Shumen	Скариди „Ванамей“	-	*Penaeus vannamei*	Columbia	Aquaculture	NA
C21	Unprocessed Frozen	Peeled	Shumen	Опашки от Бяиа скариди	-	*Litopenaeus vannamei*	Ecuador	Aquaculture	NA
C22	Unprocessed Frozen	Peeled	Stara Zagora	Скариди	-	*Litopenaeus vannamei*	Ecuador	Aquaculture	NA
C23	Unprocessed Frozen	Peeled	Dobrich	Скариди	-	*Litopenaeus vannamei*	Ecuador	Aquaculture	NA
C24	Unprocessed Frozen	Peeled	Dobrich	Скариди	-	*Litopenaeus vannamei*	Ecuador	Aquaculture	NA
C25	Unprocessed Frozen	Peeled	Dobrich	Опашки от Бяиа скариди	-	*Litopenaeus vannamei*	Ecuador	Aquaculture	NA
C26	Unprocessed Frozen	Peeled	Stara Zagora	Опашки от Бяиа скариди	-	*Litopenaeus vannamei*	Ecuador	Aquaculture	NA
C27	Unprocessed Frozen	Peeled	Shumen	Скариди	Greenland Prawns	*Pandalus borealis*	Atlantic Ocean	FAO 21	Trawl
C28	Unprocessed Frozen	Peeled	Shumen	Скариди	Greenland Prawns	*Pandalus borealis*	Atlantic Ocean	FAO 21	Trawl
C29	Unprocessed thawed	Peeled	Shumen	Скариди	Prawns	*Litopenaeus vannamei*	Pacific Ocean (Venezuela)	Aquaculture	NA
C30	Unprocessed Frozen	Peeled without head	Shumen	Бланширани скариди	-	*Litopenaeus vannamei*	India	Aquaculture	NA
C31	Unprocessed Frozen	Peeled	Shumen	Опашки от скариди	Shrimp tails	*Metapenaeus* spp., *Penaeus* spp.	Indian ocean	FAO 51,57	Trawl
C32	Unprocessed Frozen	Peeled	Stara Zagora	Опашки от скариди	Shrimp tails	*Metapenaeus* spp., *Penaeus* spp.	Indian ocean	FAO 51,57	Trawl
C33	Unprocessed Frozen	Peeled	Stara Zagora	Белени скариди	-	*Litopenaeus vannamei*	Indian ocean	FAO 51	-

C34	Unprocessed Frozen	Peeled	Stara Zagora	Белени скариди	-	*Litopenaeus vannamei*	Indian ocean	FAO 51	-
C35	Unprocessed Frozen	Whole	Stara Zagora	Белени скариди	-	*Metapenaeus affinis*	India	FAO 57	-
C36	Unprocessed Frozen	Whole	Stara Zagora	Скариди коктейлни	-	*Solenocera melantho*	China	FAO 61	Trawl
C37	Unprocessed Frozen	Whole	Stara Zagora	Гренландски скариди	-	*Pandalus borealis*	Greenland	FAO 21	Trawl
C38	Unprocessed Frozen	Squid rings	Stara Zagora	Скариди	-	*Litopenaeus vannamei*	Vietnam	Aquaculture	NA

Table S1: Labeling information of the products collected in the study NA: Not applicable

Code	Product type	Descr.	Labelled scientific name	Barcoding analysis				
				target	Length (bp)	BLAST NCBI	BOLD ID'S	Molecular id.
WF1	Unprocessed Frozen	W/o head eviscerated	*Merlangius merlangus euxinus*	COI	635	*Merlangius merlangus* 100-99.67%	*Merlangius merlangus* 100-99.21%	*Merlangius merlangus*
WF2	Unprocessed Frozen	Fillets	*Theragra chalcogramma*	COI	643	*Gadus chalcogrammus* 100-99.84%	*Gadus chalcogrammus* 100-99.84%	*Gadus chalcogrammus*
WF3	Unprocessed Chilled	Fillets	*Gadus morhua*	COI	531	***Pollachius virens* 100-99.62%**	***Pollachius virens* 100-99.62%**	***Pollachius virens***
WF4	Unprocessed Frozen	W/o head eviscerated	*Merluccius hubbsi*	COI	628	*Merluccius hubbsi* 100-99.82%	*Merluccius hubbsi* 100-99.66%	*Merluccius hubbsi*
WF5	Unprocessed Frozen	Fillets	*Theragra chalcogramma*	COI	632	*Gadus chalcogrammus* 100-99.84%	*Gadus chalcogrammus* 100-99.84%	*Gadus chalcogrammus*
WF6	Unprocessed Chilled	Fillets	*Gadus morhua*	COI	620	***Pollachius virens* 100-99.67%**	***Pollachius virens* 100-99.66%**	***Pollachius virens***
WF7	Unprocessed Frozen	Fillets – mix of fish	*Gadus morhua*	COI	637	*Gadus morhua* 100%	*Gadus morhua* 100%	*Gadus morhua*
WF8	Unprocessed Frozen	Fillets	*Theragra chalcogramma*	COI	622	*Gadus chalcogrammus* 100-99.04%	*Gadus chalcogrammus* 100-99.12%	*Gadus chalcogrammus*
WF9	Processed Frozen	Breaded precooked Fillets	*Theragra chalcogramma*	COI	626	*Gadus chalcogrammus* 100-99.14%	*Gadus chalcogrammus* 100-99.02%	*Gadus chalcogrammus*
WF10	Unprocessed Frozen	W/o head eviscerated	*Macruronus novaezelandiae*	COI	626	*Macruronus novaezelandiae* 100-99.34%	*Macruronus novaezelandiae* 100-99.34%	*Macruronus novaezelandiae*
WF11	Unprocessed Frozen	W/o head eviscerated	*Merluccius hubbsi*	COI	637	*Merluccius hubbsi* 100-99.82%	*Merluccius hubbsi* 100-99.82%	*Merluccius hubbsi*
WF12	Unprocessed Frozen	W/o head eviscerated	*Theragra chalcogramma*	COI	619	*Gadus chalcogrammus* 100-99.83%	*Gadus chalcogrammus* 100-99.83%	*Gadus chalcogrammus*
WF13	Unprocessed Frozen	Fillets	*Theragra chalcogramma*	COI	573	*Gadus chalcogrammus* 100-99.48%	*Gadus chalcogrammus* 100-99.67%	*Gadus chalcogrammus*
WF14	Unprocessed Frozen	Fillets	*Merluccius hubbsi*	COI	637	*Merluccius hubbsi* 100-99.82%	*Merluccius hubbsi* 100-99.82%	*Merluccius hubbsi*

WF15	Unprocessed Frozen	W/o head eviscerated	*Theragra chalcogramma*	COI	574	***Merluccius productus* 100-99.65%; *M. angustimanus* 99.65-99.30%: *M. gayi* 98.78%**	***Merluccius productus* 100-99.82%; *M. angustimanus* 99.82-99.30%: *M. gayi* 98.78-98-60%; *M. gayi peruanus* 98.77%**	***Merluccius productus***
				cytb	1020	***Merluccius productus* 100-99.51% *Merluccius gayi* 98.24%**		
WF16	Unprocessed Frozen	W/o head eviscerated	*Theragra chalcogramma*	COI	574	***Merluccius productus* 100-99.65%; *M. angustimanus* 99.65-99.30%: *M. gayi* 98.78%**	***Merluccius productus* 100-99.82%; *M. angustimanus* 99.82-99.30%: *M. gayi* 98.78-98-60%; *M. gayi peruanus* 98.77%**	***Merluccius productus***
				cytb	1016	***Merluccius productus* 100-99.51% *Merluccius gayi* 98.24%**	**-**	
WF17	Unprocessed Frozen	W/o head eviscerated	*Theragra chalcogramma*	COI	570	***Merluccius productus* 100-99.65%; *M. angustimanus* 99.65-99.30%: *M. gayi* 98.78%**	***Merluccius productus* 100-99.82%; *M. angustimanus* 99.82-99.30%: *M. gayi* 98.78-98-60%; *M. gayi peruanus* 98.77%**	***Merluccius productus***
				cytb	1017	***Merluccius productus* 100-99.51% *Merluccius gayi* 98.24%**	**-**	

WF18	Unprocessed Frozen	Fillets	*Theragra chalcogramma*	COI	637	*Gadus chalcogrammus* 100-99.30%	*Gadus chalcogrammus* 100-99.64%	Gadus chalcogrammus
WF19	Unprocessed Frozen	Fillets	*Theragra chalcogramma*	COI	589	***Merluccius hubbsi* 100-99.81%**	***Merluccius hubbsi* 100-99.81%**	***Merluccius hubbsi***
WF20	Unprocessed Frozen	Fillets	*Theragra chalcogramma*	COI	649	*Gadus chalcogrammus* 100-99.30%	*Gadus chalcogrammus* 100-99.64%	Gadus chalcogrammus
WF21	Unprocessed Frozen	Fillets	*Theragra chalcogramma*	COI	633	***Merluccius hubbsi* 100-99.81%**	***Merluccius hubbsi* 100-99.81%**	***Merluccius hubbsi***
WF22	Unprocessed Frozen	Fillets	*Alepocephalus bairdii*	COI	590	*Alepocephalus bairdii* 100-99.67%; *Alepocephalus rostratus* 99.66% (1 seq)	*Alepocephalus bairdii* 100-99.33%; *Alepocephalus rostratus* 99.66% (1 seq)	*Alepocephalus* sp. (species not assigned)
WF23	Unprocessed Frozen	W/o head eviscerated	*Theragra chalcogramma*	COI	645	*Gadus chalcogrammus* 100-99.22%	*Gadus chalcogrammus* 100-99.64%	*Gadus chalcogrammus*
WF24	Unprocessed Frozen	Fillets	*Theragra chalcogramma*	COI	634	*Gadus chalcogrammus* 100-99.22%	*Gadus chalcogrammus* 100-99.64%	*Gadus chalcogrammus*
WF25	Unprocessed Frozen	W/o head eviscerated	*Macruronus magellanicus*	COI	611	***Merluccius hubbsi* 100-99.81%**	***Merluccius hubbsi* 100-99.81%**	***Merluccius hubbsi***
WF26	Unprocessed Frozen	Fillets	*Pollachius virens*	COI	608	*Pollachius virens* 100-99.51%	*Pollachius virens* 100-99.67%	*Pollachius virens*
WF27	Unprocessed Frozen	Fillets	*Gadus morhua*	COI	614	*Gadus morhua* 100%	*Gadus morhua* 100%	*Gadus morhua*
WF28	Unprocessed Frozen	Fillets	*Theragra chalcogramma*	COI	625	*Gadus chalcogrammus* 100-99.84%	*Gadus chalcogrammus* 100-99.84%	*Gadus chalcogrammus*
WF29	Unprocessed Frozen	Fillets	*Pollachius virens*	COI	618	*Pollachius virens* 100-99.63%	*Pollachius virens* 100-99.67%	*Pollachius virens*
WF30	Unprocessed Frozen	Fillets	*Gadus morhua*	COI	630	*Gadus morhua* 100%	*Gadus morhua* 100%	*Gadus morhua*
WF31	Unprocessed Frozen	W/o head eviscerated	*Macruronus novaezelandiae*	COI	617	*Macruronus novaezelandiae* 100-99.22%	*Macruronus novaezelandiae* 100-99.17%	*Macruronus novaezelandiae*

WF32	Unprocessed Frozen	W/o head eviscerated	*Macruronus novaezelandiae*	COI	611	*Macruronus novaezelandiae* 100-99.20%	*Macruronus novaezelandiae* 100-99.15%	*Macruronus novaezelandiae*
WF33	Unprocessed Frozen	W/o head eviscerated	*Theragra chalcogramma*	COI	622	*Gadus chalcogrammus* 100-99.67%	*Gadus chalcogrammus* 100-99.68%	*Gadus chalcogrammus*
WF34	Unprocessed Frozen	Fillets	*Theragra chalcogramma*	COI	629	*Gadus chalcogrammus* 100-99.67%	*Gadus chalcogrammus* 100-99.68%	*Gadus chalcogrammus*
WF35	Unprocessed Frozen	Fillets	*Alepocephalus bairdii*	COI	635	*Alepocephalus bairdii* 100-99.36%; *Alepocephalus rostratus* 99.67%	*Alepocephalus bairdii* 100-99.35%; *Alepocephalus rostratus* 99.66%	*Alepocephalus* sp. Species not assigned
WF36	Unprocessed Frozen	Fillets	*Gadus morhua*	**COI**	**635**	***Pollachius virens*** **100-99.83%**	***Pollachius virens*** **100-99.67%**	***Pollachius virens***
WF37	Unprocessed Frozen	W/o head eviscerated	*Theragra chalcogramma*	COI	630	*Gadus chalcogrammus* 100-99.52%	*Gadus chalcogrammus* 100-99.69%	*Gadus chalcogrammus*
WF38	Unprocessed Frozen	W/o head eviscerated	*Theragra chalcogramma*	COI	625	*Gadus chalcogrammus* 100-99.52%	*Gadus chalcogrammus* 100-99.64%	*Gadus chalcogrammus*
WF39	Unprocessed Frozen	W/o head eviscerated	*Merluccius hubbsi*	COI	635	***Micromesistius australis*** **100-99.50%**	***Micromesistius australis*** **100-99.48%**	***Micromesistius australis***
WF40	Unprocessed Frozen	W/o head eviscerated	*Merluccius hubbsi*	COI	635	*Merluccius hubbsi* 100-99.81%	*Merluccius hubbsi* 100-99.81%	*Merluccius hubbsi*
WF41	Unprocessed Frozen	Fillets	*Theragra chalcogramma*	COI	590	*Gadus chalcogrammus* 100-99.48%	*Gadus chalcogrammus* 100-99.64%	*Gadus chalcogrammus*
WF42	Unprocessed Frozen	W/o head eviscerated	*Merluccius hubbsi*	COI	628	*Merluccius hubbsi* 100-99.60%	*Merluccius hubbsi* 100-99.67%	*Merluccius hubbsi*
WF43	Unprocessed Frozen	W/o head eviscerated	*Merlangius merlangus euxinus*	COI	625	*Merlangius merlangus* 100-99.67%	*Merlangius merlangus* 100-99.21%	*Merlangius merlangus*
WF44	Unprocessed Frozen	W/o head eviscerated	*Theragra chalcogramma*	COI	597	*Gadus chalcogrammus* 100-99.50%	*Gadus chalcogrammus* 100-99.64%	*Gadus chalcogrammus*
WF45	Unprocessed Frozen	W/o head eviscerated	*Merluccius productus*	COI	590	***Micromesistius australis*** **100-99.82%**	***Micromesistius australis*** **100-99.82%**	***Micromesistius australis***

WF46	Unprocessed Frozen	Fillets	*Merluccius productus*	COI	564	*Merluccius productus* 100-99.65%; *Merluccius angustimanus* 99.65-99.28%: *Merluccius gayi* 98.79%	*Merluccius productus* 100-99.47%; *Merluccius angustimanus* 99.82-99.29%: *Merluccius gayi* 98.93-98.57%; *Merluccius gayi peruanus* 98.69%	*Merluccius productus*
				cytb	1078	*Merluccius productus* 100-99.51% *Merluccius gayi* 98.24%	-	
WF47	Unprocessed Frozen	Fillets	*Theragra chalcogramma*	COI	582	***Merluccius hubbsi* 100-99.60%**	***Merluccius hubbsi* 100-99.67%**	**Merluccius hubbsi**
WF48	Unprocessed Frozen	Fillets	*Theragra chalcogramma*	COI	614	*Gadus chalcogrammus* 100-99.65%	*Gadus chalcogrammus* 100-99.67%	*Gadus chalcogrammus*
WF49	Unprocessed Frozen	Fillets	*Theragra chalcogramma*	COI	615	*Gadus chalcogrammus* 100-99.65%	*Gadus chalcogrammus* 100-99.67%	*Gadus chalcogrammus*
WF50	Unprocessed Frozen	Fillets	*Merluccius hubbsi*	COI	614	*Merluccius hubbsi* 100-99.60%	*Merluccius hubbsi* 100-99.67%	*Merluccius hubbsi*
WF51	Unprocessed Frozen	Fillets	*Alepocephalus bairdii*	COI	621	*Alepocephalus bairdii* 100-99.33%; *Alepocephalus rostratus* 99.31%	*Alepocephauls bairdii* 100-99.31%; *Alepocephalus rostratus* 99.30%	*Alepocephalus* sp. Species not assigned
WF52	Unprocessed Frozen	Fillets	*Theragra chalcogramma*	COI	609	*Gadus chalcogrammus* 100-99.63%	*Gadus chalcogrammus* 100-99.75%	*Gadus chalcogrammus*
WF53	Unprocessed Frozen	Fillets	*Theragra chalcogramma*	COI	603	*Gadus chalcogrammus* 100-99.60%	*Gadus chalcogrammus* 100-99.65%	Gadus chalcogrammus
WF54	Unprocessed Frozen	Fillets-	*Gadus morhua*	COI	626	*Gadus morhua* 100%	*Gadus morhua* 100%	Gadus morhua

WF55	Unprocessed Frozen	Fillets	*Pollachius virens*	COI	609	*Pollachius virens* 100-99.67%	*Pollachius virens* 100-99.67%	Pollachius virens
WF56	Processed Frozen	Fish fingers	*Theragra chalcogramma*	COI	621	*Gadus chalcogrammus* 100-99.67%	*Gadus chalcogrammus* 100-99.47%	*Gadus chalcogrammus*
WF57	Processed Frozen	Fish fingers	*Theragra chalcogramma*	COI	609	*Gadus chalcogrammus* 100-99.51%	*Gadus chalcogrammus* 100-99.47%	*Gadus chalcogrammus*
WF58	Processed Frozen	Fish fingers	*Theragra chalcogramma*	COI	610	*Gadus chalcogrammus* 100-99.67%	*Gadus chalcogrammus* 100-99.47%	*Gadus chalcogrammus*
WF59	Processed Frozen	Fish fingers	*Theragra chalcogramma*	COI	629	*Gadus chalcogrammus* 100-99.67%	*Gadus chalcogrammus* 100-99.47%	*Gadus chalcogrammus*
WF60	Processed Frozen	Fillets with brocoly	*Theragra chalcogramma*	COI	629	*Gadus chalcogrammus* 100-99.67%	*Gadus chalcogrammus* 100-99.47%	*Gadus chalcogrammus*
WF61	Processed Frozen Processed Frozen	Fish fingers	*Merluccius* spp.	COI	606	*Merluccius gayi* 100-99.83%; *Merluccius productus* 98.84-98.35%; *Merluccius angustimanus* 98.84-98.51%	*Merluccius gayi* 100-99.83%; *Merluccius gayi gayi* 100-99.83%; *Merluccius gayi peruanus* 100-99.83%; *Merluccius productus* 98.83-98.33%; *Merluccius angustimanus* 98.83-98.50%	*Merluccius gayi*
				Cytb	1019	*Merluccius gayi* 100% *Merluccius prooductus 98.23-98.13%*	-	
WF62	Processed Frozen	Breaded fillets	Theragra chalcogramma	COI	629	*Gadus chalcogrammus* 100-99.67%	*Gadus chalcogrammus* 100-99.47%	*Gadus chalcogrammus*

WF63	Processed Frozen	Fish fingers	*Merluccius* spp.	COI	584	*Merluccius productus* 100-99.49%; *Merluccius angustimanus* 100-99.30% , *M. gayi* 98.80%	*Merluccius productus* 100-99.49%; *Merluccius angustimanus* 100-99.30% , *M. gayi* 98.80%	*Merluccius productus*
				cytb	1064	*Merluccius productus*	-	
WF64	Unprocessed Frozen	Fillets	*Merluccius hubbsi*	COI	633	*Merluccius hubbsi* 100-99.83%	*Merluccius hubbsi* 100-99.81%	*Merluccius hubbsi*
WF65	Unprocessed Frozen	W/o head eviscerated	*Macruronus novaezelandiae*	COI	607	*Macruronus novaezelandiae* 100-99.16%	*Macruronus novaezelandiae* 100-99.50%	*Macruronus novaezelandiae*
WF66	Unprocessed Frozen	Fillets	*Merluccius productus*	COI	606	*Merluccius productus* 100-99.49%; *Merluccius angustimanus* 99.67-99.30% , *Merluccius gayi* 98.89-98.36%	*Merluccius productus* 100-99.49%; *Merluccius angustimanus* 100-99.30% , *Merluccius gayi* 99.00-89.36%	*Merluccius productus*
				cytb	1016	*Merluccius productus* 100-99.51% *Merluccius gayi* 98.24%	-	
WF67	Unprocessed Frozen	Fillets	*Alepocephalus bairdii*	COI	623	*Alepocephalus bairdii* 100-99.36%; *Alepocephalus rostratus* 99.67%	*Alepocephalus bairdii* 100-99.35%; *Alepocephalus rostratus* 99.66%	*Alepocephalus* sp. Species not assigned
WF68	Unprocessed Frozen	Fillets	*Alepocephalus bairdii*	COI	629	*Alepocephalus bairdii* 100-99.36%; *Alepocephalus rostratus* 99.67%	Alepocephalus bairdii 100-99.35%; *Alepocephalus rostratus* 99.66%	*Alepocephalus* sp. Species not assigned
WF69	Unprocessed Frozen	Fillets	*Theragra chalcogramma*	COI	639	*Gadus chalcogrammus* 100-99.83%	*Gadus chalcogrammus* 100-99.81%	*Gadus chalcogrammus*
WF70	Unprocessed Frozen	Fillets	*Merluccius hubbsi*	COI	646	*Merluccius hubbsi* 100-99.81%	*Merluccius hubbsi* 100-99.81%	*Merluccius hubbsi*

WF71	Unprocessed Frozen	W/o head eviscerated	*Macruronus novaezelandiae*	COI	600	*Macruronus novaezelandiae* 100-99.16%	*Macruronus novaezelandiae* 100-99.50%	*Macruronus novaezelandiae*
WF72	Unprocessed Frozen	Fillets	*Alepocephalus bairdii*	COI	590	*Alepocephalus bairdii* 100-99.36%; *Alepocephalus rostratus* 99.67%	Alepocephalus bairdii 100-99.35%; *Alepocephalus rostratus* 99.66%	*Alepocehpalus* sp. Species not assigned
WF73	Unprocessed Frozen	W/o head eviscerated	*Micromesistius australis*	COI	630	*Micromesistius australis* 100-99.50%	*Micromesistius australis* 100-99.48	*Micromesistius australis*
WF74	Unprocessed Frozen	Fillets	*Theragra chalcogramma*	COI	635	*Gadus chalcogrammus* 100-99.83%	*Gadus chalcogrammus* 100-99.81%	*Gadus chalcogrammus*
WF75	Unprocessed Frozen	Fillets	*Merluccius hubbsi*	COI	652	*Merluccius hubbsi* 100-99.81%	*Merluccius hubbsi* 100-99.81%	*Merluccius hubbsi*
WF76	Unprocessed Frozen	W/o head eviscerated	*Macruronus novaezelandiae*	COI	604	*Macruronus novaezelandiae* 100-99.20%	*Macruronus novaezelandiae* 100-99.15%	*Macruronus novaezelandiae*
WF77	Unprocessed Frozen	Fillets	Theragra chalcogramma	COI	635	*Gadus chalcogrammus* 100-99.83%	*Gadus chalcogrammus* 100-99.81%	*Gadus chalcogrammus*
WF78	Processed Frozen	Breaded fish fingers	*Theragra chalcogramma*	COI	645	***Merluccius hubbsi* 100-99.81%**	***Merluccius hubbsi* 100-99.81%**	***Merluccius hubbsi***
WF79	Processed Frozen	Breaded fillets	*Theragra chalcogramma*	COI	600	***Merluccius gayi* 100-99.83%; *Merluccius productus* 98.84-98.35%; *Merluccius angustimanus* 98.84-98.51%**	***Merluccius gayi* 100-99.83%; *Merluccius gayi gayi* 100-99.83%; *Merluccius gayi peruanus* 100-99.83%; *Merluccius productus* 98.83-98.33%;*Merluccius angustimanus* 98.83-98.50%**	***Merluccius gayi***
				Cytb	974	***Merluccius gayi* 100% *Merluccius productus* 98.13%**	**-**	

WF80	Unprocessed Frozen	Fillets	Theragra chalcogramma	COI	633	*Gadus chalcogrammus* 100-99.69%	*Gadus chalcogrammus* 100-99.69%	*Gadus chalcogrammus*
WF81	Unprocessed Frozen	Breaded fillets	*Merluccius* sp	COI	607	*Merluccius gayi* 100-99.83%: *Merluccius productus* 98.85-98.52%; *Merluccius angustimanus* 98.52%	*Merluccius gayi* 100-99.83%; *Merluccius gayi gayi* 100%; *Marluccius gayi peruanus* 100%; *Merluccius productus* 98.80%	*Merluccius gayi*
				Cytb	1019	*Merluccius gayi* 100% *Merluccius productus* 98.13%	-	
WF82	Processed Frozen	Breaded fillets	*Merluccius* sp.	COI	611	*Merluccius gayi* 100-99.83%: *Merluccius productus* 98.85-98.52%; *Merluccius angustimanus* 98.52%	*Merluccius gayi* 100-99.83%; *Merluccius gayi gayi* 100%; *Merluccius gayi peruanus* 100%; *Merluccius productus* 98.80%	*Merluccius gayi*
				Cytb	970	*Merluccius gayi*	-	
WF83	Processed Frozen	Breaded fillets	*Theragra chalcogramma*	COI	651	*Gadus chalcogrammus* 100-99.69%	*Gadus chalcogrammus* 100-99.69%	*Gadus chalcogrammus*
WF84	Processed Frozen	Breaded fillets	*Theragra chalcogramma*	COI	651	*Gadus chalcogrammus* 100-99.69%	*Gadus chalcogrammus* 100-99.69%	*Gadus chalcogrammus*
WF85	Unprocessed Frozen	W/o head eviscerated	*Merlangius merlangus euxinus*	COI	643	*Merlangius merlangus* 100-99.67%	*Merlangius merlangus* 100-99.22%	*Merlangius merlangus*
WF86	Unprocessed Frozen	W/o head eviscerated	*Merluccius hubbsi*	COI	601	*Merluccius hubbsi* 100-99.81	*Merluccius hubbsi* 100-99.81%	*Merluccius hubbsi*
WF87	Unprocessed Frozen	Fillets	*Merluccius hubbsi*	COI	601	*Merluccius hubbsi* 100-99.81	*Merluccius hubbsi* 100-99.81%	*Merluccius hubbsi*
WF88	Unprocessed Frozen	Fillets	*Theragra chalcogramma*	COI	645	*Gadus chalcogrammus* 100-99.69%	*Gadus chalcogrammus* 100-99.69%	*Gadus chalcogrammus*

WF89	Processed Frozen	Fish fingers	*Macruronus novaezelandiae*	COI	606	*Macruronus novaezelandie* 100-99.24%	*Macruronus novaezelandiae* 100-99.33%	*Macruronus novaezelandiae*
WF90	Unprocessed Frozen	W/o head eviscerated	*Merluccius hubbsi*	COI	601	*Merluccius hubbsi* 100-99.81	*Merluccius hubbsi* 100-99.81%	*Merluccius hubbsi*
WF91	Unprocessed Frozen	W/o head eviscerated	*Merluccius australis*	COI	643	*Merluccius australis* 100-99.67%	*Merluccius australis* 100-99.82%	*Merluccius australis*
WF92	Unprocessed Frozen	Fillets	*Alepocephalus bairdii*	COI	582	*Alepocephalus bairdii* 100-99.67%; *Alepocephalus rostratus* 99.66% (1 seq)	*Alepocephalus bairdii* 100-99.33%; *Alepocephalus rostratus* 99.66% (1 seq)	*Alepocephalus* sp. Species not assigned
WF93	Processed Frozen	Fish fingers	*Theragra chalcogramma*	COI	631	*Gadus chalcogrammus* 100-99.69%	*Gadus chalcogrammus* 100-99.69%	*Gadus chalcogrammus*
WF94	Unprocessed Frozen	W/o head eviscerated	*Macruronus novaezelandiae*	COI	600	*Macruronus novaezelandiae* 100-99.24%	*Macruronus novaezelandiae* 100-99.33%	*Macruronus novaezelandiae*
WF95	Unprocessed Frozen	Fillets	*Merluccius productus*	COI	643	*Merluccius productus* 100-99.84%; *Merluccius angustimanus* 99.69-99.38%; *Merluccius gayi* 99.02%	*Merluccius productus* 100-99.84%; *Merluccius angustimanus* 100-99.27%; *M. gayi* 99.06-98.90%,*M. gayi gayi* 98.90-98.73%;	*Merluccius productus*
				cytb	1020	*Merluccius productus* 100-99.51% *Merluccius gayi* 98.24%	-	
WF96	Unprocessed Frozen	Fillets	*Theragra chalcogramma*	COI	609	*Gadus chalcogrammus* 100-99.83%	*Gadus chalcogrammus* 100-99.83%	*Gadus chalcogrammus*
WF97	Unprocessed Frozen	Fillets	*Alepocephalus bairdii*	COI	574	*Alepocephalus bairdii* 100-99.30%; *Alepocephalus rostratus* 99.60%	*Alepocephalus bairdii* 100-99.30%; *Alepocephalus rostratus* 99.66%	*Alepocephalus* sp. Species not assigned

WF98	Unprocessed Frozen	Fillets	*Theragra chalcogramma*	COI	519	*Gadus chalcogrammus* 100-99.79%	*Gadus chalcogrammus* 100-99.81%	*Gadus chalcogrammus*
WF99	Unprocessed Frozen	W/o head eviscerated	*Theragra chalcogramma*	COI	631	*Gadus chalcogrammus* 100-99.83%	*Gadus chalcogrammus* 100-99.83%	*Gadus chalcogrammus*
WF100	Unprocessed Frozen	W/o head eviscerated	*Micromesistius australis*	COI	641	*Micromesistius australis* 100-99.53%	*Micromesistius australis* 100-99.30%	*Micromesistius australis*
MC1	Processed Marinated, canned	Tentacle	*Dosidicus gigas*	COI	600	*Dosidicus gigas* 100-99.03%	*Dosidicus gigas* 100-99.18%	*Dosidicus gigas*
MC2	Processed precooked Ready to eat	Slices	*Sepia* sp.	COI	556	*Sepia recurvirostra* 100-89.85%; *Sepia madokai* 99.65% (1 seq, unpublished paper)	*Sepia recurvirostra* 100-89.87%	*Sepia recurvirostra*
MC3	Processed precooked Ready to eat	Slices	*Octopus vulgaris*	COI	643	*Octopus vulgaris* 100-98.89%	*Octopus vulgaris* 100%	*Octopus vulgaris*
MC4	Unprocessed Frozen	Tentacles	*Dosidicus gigas*	COI	616	*Dosidicus gigas* 100-99.03%	*Dosidicus gigas* 100-99.18%	*Dosidicus gigas*
MC5	Unprocessed Frozen	Whole peeled eviscerated	*Dosidicus gigas*	COI	616	*Dosidicus gigas* 100-99.03%	*Dosidicus gigas* 100-99.18%	*Dosidicus gigas*
MC6	Unprocessed Frozen	Whole peeled eviscerated	*Ommastrephes bartramii*	COI	618	***Dosidicus gigas*** **100-99.51%**		
